# An automated do-it-yourself system for dynamic stem cell and organoid culture in standard multi-well plates

**DOI:** 10.1016/j.crmeth.2022.100244

**Published:** 2022-07-01

**Authors:** Julia Tischler, Zoe Swank, Hao-An Hsiung, Stefano Vianello, Matthias P. Lutolf, Sebastian J. Maerkl

**Affiliations:** 1 Laboratory of Biological Network Characterization, Institute of Bioengineering, School of Engineering, École Polytechnique Fédérale de Lausanne (EPFL), Lausanne, 1015 Vaud, Switzerland; 2 Brigham and Women’s Hospital, Harvard Medical School, Boston, MA 02115, USA; 3 Laboratory of Stem Cell Bioengineering, Institute of Bioengineering, School of Life Sciences and School of Engineering, École Polytechnique Fédérale de Lausanne (EPFL), Lausanne, 1015 Vaud, Switzerland; 4 Roche Institute for Translational Bioengineering (TB), Pharma Research and Early Development (pRED), F. Hoffman-La Roche Ltd, Basel, Switzerland

**Keywords:** fully automated system for complex mammalian cell culture, mouse embryonic stem cell culture, mouse 3D gastruloid culture, organoid culture, microfluidic control, medium formulation in real time, mammalian cell culture under dynamically changing medium compositions, programmable cell culture system, fully automated media exchanges, complex cell culture in standard multi-well plates

## Abstract

We present a low-cost, do-it-yourself system for complex mammalian cell culture under dynamically changing medium formulations by integrating conventional multi-well tissue culture plates with simple microfluidic control and system automation. We demonstrate the generation of complex concentration profiles, enabling the investigation of sophisticated input-response relations. We further apply our automated cell-culturing platform to the dynamic stimulation of two widely employed stem-cell-based *in vitro* models for early mammalian development: the conversion of naive mouse embryonic stem cells into epiblast-like cells and mouse 3D gastruloids. Performing automated medium-switch experiments, we systematically investigate cell fate commitment along the developmental trajectory toward mouse epiblast fate and examine symmetry-breaking, germ layer formation, and cardiac differentiation in mouse 3D gastruloids as a function of time-varying Wnt pathway activation. With these proof-of-principle examples, we demonstrate a highly versatile and scalable tool that can be adapted to specific research questions, experimental demands, and model systems.

## Highlights

• Microfluidic control and system automation facilitate complex mammalian cell culture
• Integration into conventional multi-well tissue culture plates offers maximum flexibility
• Automated DIY cell-culturing platform can be tailored to specific experimental demands
• Automated medium-switch experiments unravel parameters guiding early mammalian development

## Motivation

*In vitro* models, in particular emerging 3D cell culture systems such as gastruloids and organoids, can offer invaluable insight into developmental programs that are largely inaccessible for study *in vivo*, aid in deciphering molecular pathways that have gone awry in disease, and hold promise for paving the way toward personalized precision medicine. However, there is an unmet need in the availability of affordable, simple-to-implement, and straightforward-to-use tools that enable complex mammalian cell culture. With the development of a do-it-yourself system, we present a technology enabling the fully automated culture and dynamic stimulation of cells and cellular aggregates in conventional multi-well plates.

## Graphical abstract

**Figure:**
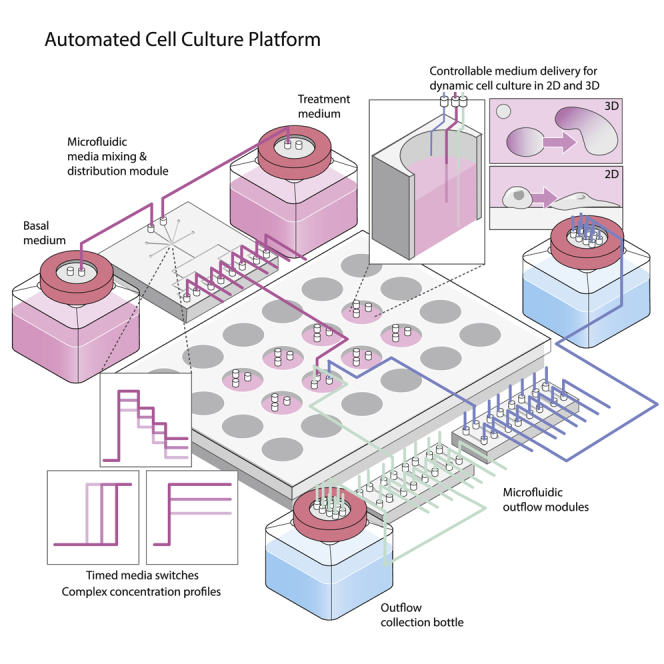

## Teaser

By integrating standard multi-well tissue culture plates with microfluidic control and system automation, Tischler et al. engineer arrays of individually addressable and programmable cell culture chambers. This low-cost, do-it-yourself cell-culturing platform enables the fully automated, complex culture and dynamic stimulation of mammalian cells, cellular aggregates, tissue explants, and embryos.

## Introduction

*In vitro* cell culture technologies provide powerful tools for comprehensively exploring the principles underlying developmental programs during mammalian embryogenesis, as well as disease onset and progression (Kim et al., 2020; Simunovic and Brivanlou, 2017). Conventionally, mammalian cell culture is performed in batch and involves predominantly manual medium exchange and sub-culturing routines, conducted in daily intervals at best (Masters and Stacey, 2007; Mulas et al., 2019). However, manual cell-culturing techniques are cumbersome and prone to operator error (Niepel et al., 2019), making it difficult to achieve precisely controlled processes, and restricting the scope and complexity of possible investigations, such as the impact of different types, doses, and temporal stimulation profiles of cytokines, drugs, or small-molecule modulators, or combinations thereof, on cell fate. Furthermore, the build-up of cell-secreted factors and excreted metabolic waste products in standard batch cultures presents a challenge to precisely controlling cellular behavior and cell fate decisions, and to quantitatively predicting cell fate outcomes. Critically, because of extremely limited temporal control over medium composition, conventional manual batch culture techniques largely preclude investigation of cellular behavior in response to complex, dynamically changing environments. Notably, cellular decision-making, cell fate specification, and developmental programs are guided by intricate, temporally varying signaling dynamics (Junkin et al., 2016; Mondragón-Palomino et al., 2011; Sorre et al., 2014; Zhang et al., 2019). Thus, straightforward technologies for automatically supplying time-varying extra-cellular stimuli to cell cultures, and for administering complex drug treatment schemes, would open avenues for improved control and defined modulation of cell fate specification programs, with significant implications for tissue engineering and regenerative and personalized medicine. Well-defined, precisely controlled, and highly consistent cell culture conditions are also highly desired for cell-based therapies and biomedical applications. Currently, however, simple and accessible tools are lacking that enable fully automated medium exchange routines and the delivery of complex, dynamic inputs to a broad array of biologically relevant *in vitro* cell culture models in 2D and 3D.

The latest developments in microfluidic technologies for cell biology applications present a major milestone (Gómez-Sjöberg et al., 2007). Sophisticated microfluidic devices have been engineered for the distribution of combinatorial and time-varying signals to dozens of individually addressable miniature cell culture chambers and enabled the exploration of complex cellular behaviors in response to dynamic modulation of the cell culture environment at an unparalleled precision and scale (Junkin et al., 2016; Sorre et al., 2014; Zhang et al., 2019). However, the small size of microfluidic devices and the challenges of cell loading and subsequent recovery restrict the number of cells available for analysis and downstream applications, particularly in regard to the investigation of emerging 3D cell culture models and tissue explants. A high-throughput microfluidic platform was recently reported for the automated culture and drug treatment of tumor organoids (Schuster et al., 2020). Overlaying a custom microwell array with microfluidic channels enabled combinatorial and sequential drug screening in conjunction with real-time imaging and subsequent harvesting of organoid cultures for further analyses. However, the required know-how, expertise, and design complexity of state-of-the-art microfluidic cell-culturing devices limit adaptation and modification by non-expert laboratories. Lately, a simpler-to-establish microfluidic system was presented for the culture and pulsed stimulation of primary mouse tissue in *ex vivo* cultures (Sanchez et al., 2021; Sonnen et al., 2018; van Oostrom et al., 2021). Neither culture system, however, enables the exploration of how complex stimulation profiles and signaling dynamics, such as step-function concentration changes or oscillatory pulses, impact cellular systems, while necessitating culturing of cellular aggregates in polydimethylsiloxane (PDMS) chambers.

Additionally, despite the benefits of state-of-the-art microfluidic technologies and their applications to complex mammalian cell culture, the few commercially available microfluidic systems are severely limited in scope. Ibidi offers basic technology at the intersection of fluidics and mammalian cell culture. Supporting *in vitro* cell culture under controlled flow conditions in conjunction with live-cell imaging, ibidi’s channel slide and pump systems enable investigations of the impact of mechanical forces on cellular behavior and morphology, offer optimal supply of nutrients and gases to cells cultured in 3D matrices, and defined medium exchanges. Similarly, EBERS and KDBIO distribute microfluidic devices, cell culture modules, and bioreactors, respectively, for the culture of mammalian cells under flow in order to simulate shear stress and to emulate physiological environments. Despite providing remarkable tools for exploring the effects of complex cell culture conditions that are unattainable to mimic with conventional manual cell-culturing approaches, these systems lack dynamic control over medium conditions, and offer no, or only extremely limited, multiplexing capabilities. Merck’s CellASIC ONIX microfluidic platform enables the culture of bacterial, yeast, and mammalian cells under user-defined, changing medium inputs, flow rates, and environmental (temperature and gas) control. The imaging-compatible mammalian cell culture plates facilitate direct observation of cellular behavior in four imaging chambers within a microfluidic chip, and comparison of up to four medium conditions in parallel. The platform supports long-term (typically 3 days) live-cell imaging experiments of adherent cells, with user-defined solution exchanges. However, the system’s capabilities for the culture of cellular aggregates, such as organoids or spheroids, are limited (3- to 6-h experiments only, without environmental control). While the platform offers protocols for cell fixation “on-demand” and automated immune-staining, the difficulty of recovering cells from the microfluidic chambers presents a major drawback of the system and precludes downstream analyses. Facilitating the culture of human cells in defined microenvironments on microfluidic devices, EMULATE, Inc., offers sophisticated “organ-on-a-chip” technology to model human organs and provide insight into disease and for drug development and efficacy testing. A programmable culture module enables the fully automated, parallel culture of up to 12 “organ-chips” under defined flow rates. However, the system does not support organ-chip culture under dynamically changing medium formulations and stimulation profiles, respectively.

Although automated liquid-handling robots could, in principle, be employed to perform challenging medium exchange routines at a high throughput, enabling cell culture under complex, time-varying medium compositions, with little optimization and development time, their bulky size, and high production and maintenance costs have limited their use in cell culture applications to very large industrial settings at best. Critically, such systems are not readily compatible with concurrent time-lapse microscopy.

Recently, eVOLVER, a do-it-yourself (DIY) platform, was presented for the automated and dynamic control of yeast and bacterial culture conditions (Wong et al., 2018). The system is composed of customizable “smart sleeves,” machined aluminum tubes equipped with sensors and actuators, to hold and interface with individual culture vials, and a hardware-software interface. Milliflluidic modules, formed by a silicone rubber membrane held between two sheets of laser-etched plastic, patterned with fluidic channels, accomplish complex fluidic manipulations, such as multiplexed medium routing for dynamic-medium formulation, and liquid transfers between separate culture vessels. The modular design of the system enables the rapid and low-cost scaling and re-configuration to fit experimental demands. However, no comparable platforms exist for the automated culture of mammalian cells under temporally changing medium conditions.

Here, we present the design, development, and validation of a low-cost, simple-to-implement and use proof-of-concept DIY system to perform complex mammalian cell culture experiments in conventional multi-well plates. In conjunction with system automation and microfluidic control, our cell-culturing platform offers precise, temporal regulation and modulation of cell culture conditions, through fully automated medium exchange routines and dynamically changing media compositions, formulated in real-time, in eight individually addressable culture chambers in parallel. By integrating into any commercially available multi-well tissue culture plate, our system is flexible and versatile, offering unprecedented opportunities for investigating complex input-response relations in a plethora of cellular and developmental systems.

In its DIY nature and modular design, our automated cell-culture platform (ACCP) supports straightforward modification and adaptation by non-expert users.

We demonstrate the potential of our system through the formulation of complex, temporally changing concentration profiles. As a proof-of-principle, we apply the system to track cell fate commitment along the developmental trajectory toward mouse epiblast fate *in vitro*, and examine symmetry-breaking, germ layer formation, and cardiac potential of mouse 3D gastruloids in response to time-varying stimulatory pulses. Together, we show that our fully automated DIY system provides a robust and controlled environment for the culture and differentiation of sensitive primary cells, and of emerging 3D *in vitro* model systems.

## Results

### Development of an automated mammalian cell-culture system

**Figure 1:**
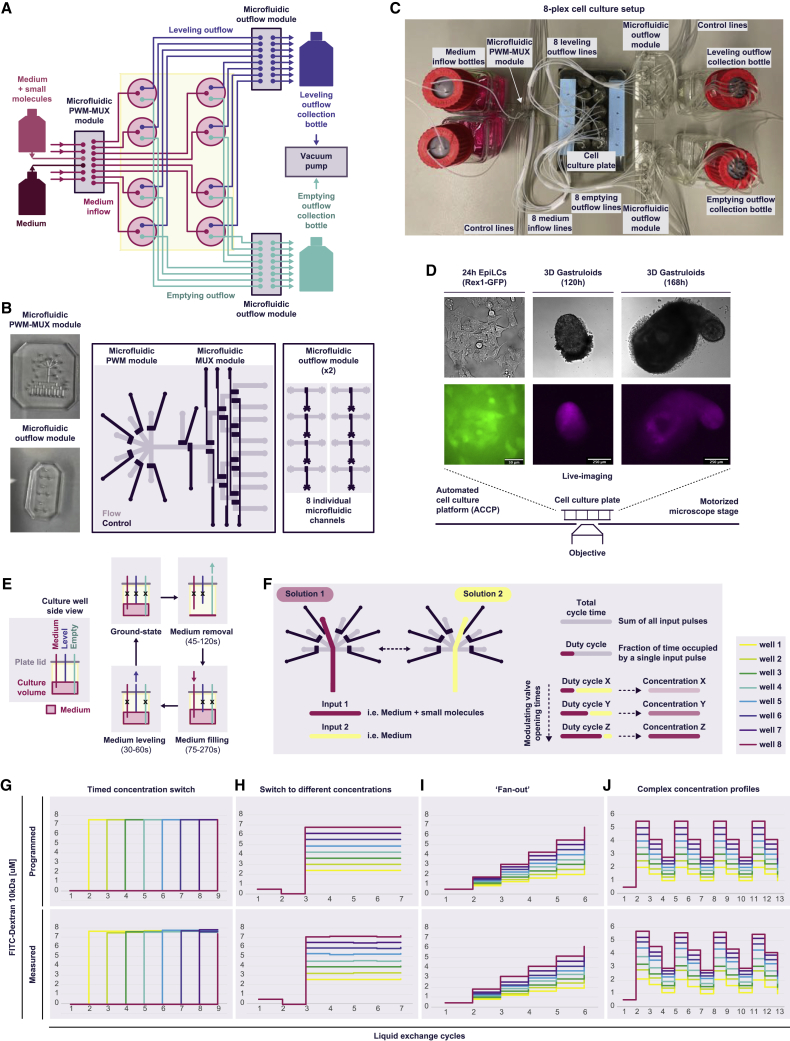
DIY platform for the fully automated multiplexed culture and dynamic stimulation of mammalian cells (A) Medium is routed through microfluidic modules, and an engineered plate lid. The re-usable, DIY fluidic control lid mediates addition and removal of liquid to each culture well. Fluidic interconnects for medium inflow (“medium," in magenta), level setting (“level," in turquoise), and outflow (“empty," in blue) are depicted. The integrated microfluidic pulse width modulation and multiplexing (PWM-MUX) module enables selection from a maximum of six different medium inputs, dynamic on-chip medium formulation, and dispensing to eight individually addressable culture wells. Outflow medium is routed through single flow channels into waste collection bottles attached to a vacuum pump. (B) Microfluidic control modules are fabricated from PDMS by standard two-layer soft lithography. (C) 8-plex cell culture setup, corresponding to the schematic in (A). (D) Mounting of the setup onto an automated microscope stage enables live-cell imaging. (E) A full medium exchange cycle is illustrated; x denotes closed channels. (F) Schematic for the PWM-mediated formulation of medium compositions. (G–J) ACCP-mediated generation of time-varying concentration profiles through the pre-programed on-chip mixing of a 7.5 μM fluoresceinisothiocyanate (FITC)-dextran 10kDa solution and medium, and liquid routing to eight individual culture wells.

We engineered a proof-of-concept system for a low-cost, DIY automated cell-culturing platform (ACCP), composed of a conventional multi-well tissue culture plate, a simple and re-usable plate lid for achieving liquid exchanges in individual culture wells, and low-complexity, easy-to-fabricate microfluidic modules for fluidic control, dynamic medium formulation, and dispensing (Figures 1A–1C). The system is controlled by a simple pneumatic setup and software (Brower et al., 2018; White and Streets, 2018). Integration into any multi-well tissue culture plate of choice offers maximum flexibility and supports the multiplexed culture of adherent cells in 2D and cellular aggregates in 3D (such as embryoids, gastruloids, organoids, spheroids, and tissue explants). The use of standard culture wells enables simple cell loading and recovery for downstream cell culture applications and molecular characterization, ensures compatibility with cell-specific substrates, and facilitates the rapid re-configuration (i.e., to larger or smaller well sizes, specific surface coatings, internal structures [such as microwells], different cell types or model systems, etc.) to meet specific experimental needs.

The re-usable fluid exchange lid can be easily fabricated by non-experts. Access ports are drilled into a standard polystyrene lid and fitted with tubing entering individual culture chambers from the top (Figures 1C and S1). For each well, fluidic interconnects are assembled for medium inflow, outflow, and setting of the cell culture volume levels. The length of the tubing reaching into the wells determines the upper medium level, and thus the culture volume, and the volume of medium that is replaced during each liquid exchange cycle. When required, additional interconnects can be integrated to allow direct CO_2_ perfusion into the head-space of individual culture wells. We engineered fluidic control lids for custom 24-well polystyrene plates (nunc, Life Technologies), imaging-compatible 24-well plates (ibidi u-plates), and Gri3D hydrogel microwell array 24-well plates (SUN bioscience). Fluid exchange lids were configured to a final culture volume of approximately 750 μL per standard 24-well (nunc, ibidi u-plates), and about 1,330 μL for the Gri3D hydrogel microwell arrays (SUN bioscience), with near-complete liquid removal achieved by outflow tubing reaching to the bottom of the well, fitted with a kinked metal pin and directed at the edge of the well to minimize aspiration of cells during full emptying cycles.

Liquid flow is controlled by low-complexity microfluidic modules, fabricated by standard two-layer soft lithography from PDMS (Kim et al., 2012) (Figure 1B). Previous DIY methods have shown that fluidic devices of similar or higher complexity can be used in such applications without limiting adaptability of the approach (Wong et al., 2018), and the fluid manipulations required by the ACCP can be achieved with these or a variety of other microfluidic technologies. Operation of the microfluidic modules is mediated through generic solenoid valves controlled by a LabVIEW script. Medium flow is pressure driven and supports facile tuning of the flow rate (Figure S1). Pressurizing the medium reservoirs with 5% CO_2_ enables culturing of CO_2_-dependent cells, such as embryonic stem cells (ESCs) (Mulas et al., 2019). Medium level setting and outflow are achieved by vacuum aspiration, with liquid routed into waste collection bottles (Figures 1A and 1C). Placing the setup onto a motorized microscope stage enclosed in a temperature-controlled chamber offers the additional benefit of concurrent real-time imaging (Figures 1D and S1).

Fully automated medium exchange cycles are achieved through programmed opening of a single microfluidic outflow channel, followed by fresh medium dispensing and setting of the culture volume through the operation of an efflux channel that controls the medium level (Figure 1E). At an inflow pressure of 5 psi, one complete cycle consisting of emptying, re-filling, and leveling of a single 24-well culture well required less than 3 min. The ACCP is designed to achieve two fully automated modes of operation: a cell culture mode, performing complete medium exchanges at user-defined intervals, and an integrated dynamic mode, enabling timed medium switches, and complex real-time medium formulation via microfluidic pulse width modulation (PWM) (Ainla et al., 2009; Azizi and Mastrangelo, 2008; Cao et al., 2010; Woodruff and Maerkl, 2018; Zhang et al., 2010).

Upstream incorporation of a microfluidic PWM module enables dynamic formulation of input solutions by alternate opening and closing of inflow channels drawing liquid from distinct medium sources (Figures 1F and S1). Temporally modulating the flow times of specific medium inputs generates different medium compositions and concentration profiles. A microfluidic multiplexing (MUX) module (Thorsen et al., 2002) downstream of the PWM module offers the ability to independently address eight culture wells, each with individually customizable, time-varying medium formulations.

Wash cycles (STAR Methods) integrated within the dynamic operation mode of the ACCP clear the PWM-MUX module and the connecting tubing of medium formulations from the directly preceding PWM cycles. Implementing wash steps in conjunction with complete medium removal from the culture wells facilitates instantaneous medium changes and thus the discrete switching to newly formulated medium compositions. Running the setup on a motorized microscope stage supports validation of PWM-generated and dynamically modulated medium compositions through the real-time tracking of supplemented fluorescent-dye tracers and direct observation of cellular and organismal behavior.

To demonstrate the capabilities of our DIY system to precisely formulate pre-programmed medium compositions in real-time, route liquid to specific culture chambers, and perform fully automated medium exchange cycles at desired intervals, we designed several distinct fluidic routines for complex operational modes (Figures 1G–1J). First, we devised fluidic operations for timed solution exchanges (Figure 1G). This operational mode enables systematic investigations of cellular decision-making and commitment during cell fate specification programs or the dependence of cell fate outcomes on the precise timing and duration of a stimulatory pulse. Second, we designed fluidic routines for establishing specific concentrations among individual culture wells (Figure 1H). This functionality is powerful for probing concentration-dependent effects, identifying optimal concentrations of specific cytokines or signaling or pharmacological modulators, and refining cell culture protocols. Third, we programmed complex patterns, such as stepwise increases in concentrations (Figure 1I) and dynamic ramping up and down to specified concentrations (Figures 1J and S1). These sophisticated dynamic fluidic routines could enable cell fate determination as a function of the speed and frequency of cytokine presentation (Junkin et al., 2016; Sorre et al., 2014), entrainment of signaling pathway dynamics, and defined perturbations of molecular clocks through the delivery of complex oscillatory inputs (Mondragón-Palomino et al., 2011). This mode could also be used to emulate intricate pharmacodynamic profiles for toxicology studies. We tested the system by generating desired output concentrations through dynamic on-chip mixing of a fluorescein solution and buffer, drawn from two separate inlets of the PWM module. The measured concentration profiles, derived from quantitative analyses of fluorescent time-lapse image series, closely matched the programmed patterns, validating the capability of the ACCP to perform intricate fluidic manipulations and to generate pre-programmed, dynamically changing concentration gradients with high precision and robustness (Figures 1G–1J and S1).

### Tracking cell fate commitment through automated medium switches

**Figure 2:**
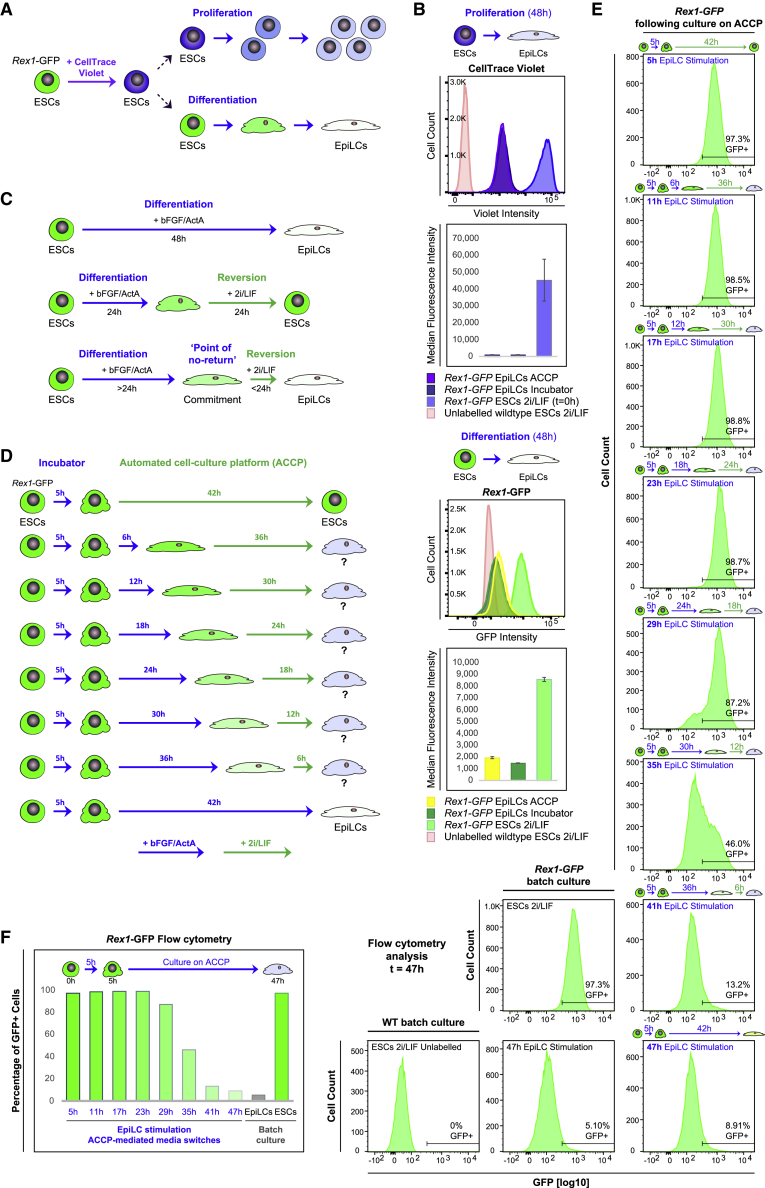
Cell fate commitment along the developmental trajectory toward epiblast fate (A and B) (A) Assessing cellular proliferation and differentiation during *in vitro* conversion of naive mouse ESCs into EpiLCs. (B) Flow cytometry analysis of CellTrace Violet-labeled *Rex1*-GFP reporter cells following 48 h of EpiLC stimulation under hourly ACCP-mediated medium exchange cycles and conventional batch culture. CellTrace Violet-stained *Rex1*-GFP reporter ESCs (t = 0) are shown as a reference. Graphs represent averages from two independent biological experiments. Error bars denote ±SE. Representative flow cytometry profiles are shown. (C–F) Investigating cell fate commitment during the ESC-to-EpiLC transition through timed medium switch experiments. (D) Culture schemes and fluidic routines employed. (E and F) Flow cytometry-based quantification of the fraction of *Rex1*-GFP positive (GFP+) cells after different durations of EpiLC induction. bFGF, basic fibroblast growth factor; ActA, activin A.

We next applied our system to the automated culture of a widely employed stem-cell-based 2D *in vitro* cell culture model for mammalian epiblast development: the transition from naive pluripotency into primed epiblast-like cell fates (Hayashi et al., 2011). Using a reporter assay in conjunction with a dye dilution approach, we assessed growth and directed differentiation of naive mouse ESCs under fully automated, hourly medium exchange cycles (Figures 2A, 2B, and S1). After labeling with CellTrace Violet as a means to track cell division, we stimulated the *in vitro* conversion of a reporter ESC line expressing *Rex1*-GFP, which marks the naive pluripotent state (Kalkan et al., 2017; Wray et al., 2011), into primed epiblast-like cells (EpiLCs) (Figure 2A), using a well-established and robust induction protocol (Hayashi et al., 2011). Flow cytometry-based quantification of CellTrace Violet dye levels after 48 h of EpiLC stimulation confirmed similar proliferation rates of cells cultured on the ACCP and cells grown under conventional batch culture conditions in a tissue culture incubator (Figure 2B). Likewise, 48 h of EpiLC induction led to a loss of *Rex1*-GFP expression in both conditions (Figure 2B), supporting the efficient directed differentiation of ESCs (Hayashi et al., 2011) on the ACCP.

Aa a proof-of-principle, we then applied the ACCP to investigate cell fate commitment along the developmental trajectory toward epiblast fate (Figure 2C). Cells continue to express *Rex1* upon transfer into naive pluripotency-promoting (2i/LIF) culture conditions (Ying et al., 2008) within 24 h of EpiLC stimulation (Murakami et al., 2016). However, this potential is largely irreversibly lost by 48 h of stimulation (Murakami et al., 2016; Tischler et al., 2019). To systematically examine the point at which cells become committed to continue their trajectory toward epiblast fate during the ESC-to-EpiLC conversion, we designed a fluidic routine for automated medium switching, where EpiLC-inducing medium was sequentially replaced (in 6-h intervals) by 2i/LIF medium in each of the eight parallel culture wells, with full medium exchange cycles occurring every 2 h (Figure 2D). Concurrent time-lapse imaging confirmed medium switches at desired intervals, cellular growth, and morphological changes (Figure S2, and Video S1 for an example). We performed flow cytometry analyses as endpoint measurements after a total of 47 h of culture (Figures 2E, 2F, and S2). Quantification of *Rex1*-GFP reporter expression suggested a first change in pluripotent potential as early as 29 h of culture in EpiLC-inducing conditions, when a subset of cells no longer sustained a *Rex1*-GFP positive, ESC-like state upon reversion into 2i/LIF conditions (Figures 2E and 2F). The cells’ potential to retain an ESC-like state declined rapidly upon continued culture in EpiLC-inducing medium. Within 35 h of culture, a majority of cells had acquired a *Rex1*-GFP-negative state. The fraction of cells that had retained an ESC-like state, with continued *Rex1*-GFP expression, was greatly diminished by 41 h of EpiLC stimulation. Thus, following an extended period of sustained pluripotency, a sharp transition in developmental potential appeared to occur between approximately 29 and 35 h of culture in EpiLC-inducing conditions, rendering cells largely irresponsive to naive pluripotency-promoting signals. This “developmental point of no return” likely underlies key epigenetic and metabolic changes that govern the transition into epiblast fate (Buecker et al., 2014; Hayashi et al., 2011; Murakami et al., 2016; Tischler et al., 2019; Zylicz et al., 2015).

Video S1. 42-h time-lapse acquisition (DIA images) of *Rex1*-GFP reporter cells during reversion into naive pluripotency-promoting (2i/LIF) conditions on the ACCP, following an initial 5 h of EpiLC-stimulation, related to Figure 2 Time resolution, 1 h. Scalebar, 100μm.

Results from our automated, timed medium-exchange experiments along the developmental trajectory toward epiblast fate validated findings on the timing and irreversibility of the exit from naive pluripotency, recently reported by Strawbridge and colleagues (Strawbridge et al., 2020). Through in-depth cellular and functional characterization upon release of ESCs from 2i culture into N2B27 medium (without growth factor stimulation), Strawbridge et al. revealed a rapid decline of *Rex1*-GFP reporter expression, following an initially variable lag-phase at the level of the individual cells, and directly preceding irreversible exit from ground-state pluripotency.

### Time-varying chiron stimulation of 3D gastruloids in microwell arrays

As a further proof of concept, and to demonstrate the versatility and applicability of the ACCP to a diverse set of model systems, we turned to mouse 3D gastruloids, an emerging *in vitro* model for early mammalian post-implantation development (Steventon et al., 2021; van den Brink and van Oudenaarden, 2021). 3D gastruloids, aggregates of pluripotent stem cells, self-assemble and faithfully recapitulate fundamental processes that guide mammalian embryogenesis and form multiple derivatives of the three germ layers (Beccari et al., 2018; van den Brink et al., 2014). Transient activation of the Wnt-signaling pathway, mediated through inhibition of GSK3 via pulsed delivery of the Wnt agonist Chiron (Chir) between 48 and 72 h after gastruloid assembly, is key for inducing symmetry breaking in the spherical aggregates, gastruloid elongation, and further development (Beccari et al., 2018; van den Brink et al., 2014).

Aiming to acquire a better understanding of how the duration of Wnt pathway stimulation impacts embryonic development, we set out to systematically vary the length of the Chir pulse and comprehensively examine symmetry-breaking, germ layer specification, and the formation of cardiac tissues in multiple mouse 3D gastruloids cultured in parallel. Complementing previous, coarse-grained investigations on the impact of Chir pulse timing on gastruloid fate, performed by manually administering 24-h pulses in 24-h “sliding” windows from 24 to 72 h after gastruloid aggregation (Turner et al., 2017), we sought to deliver Chir pulses with a higher time resolution of 4-h intervals.

**Figure 3:**
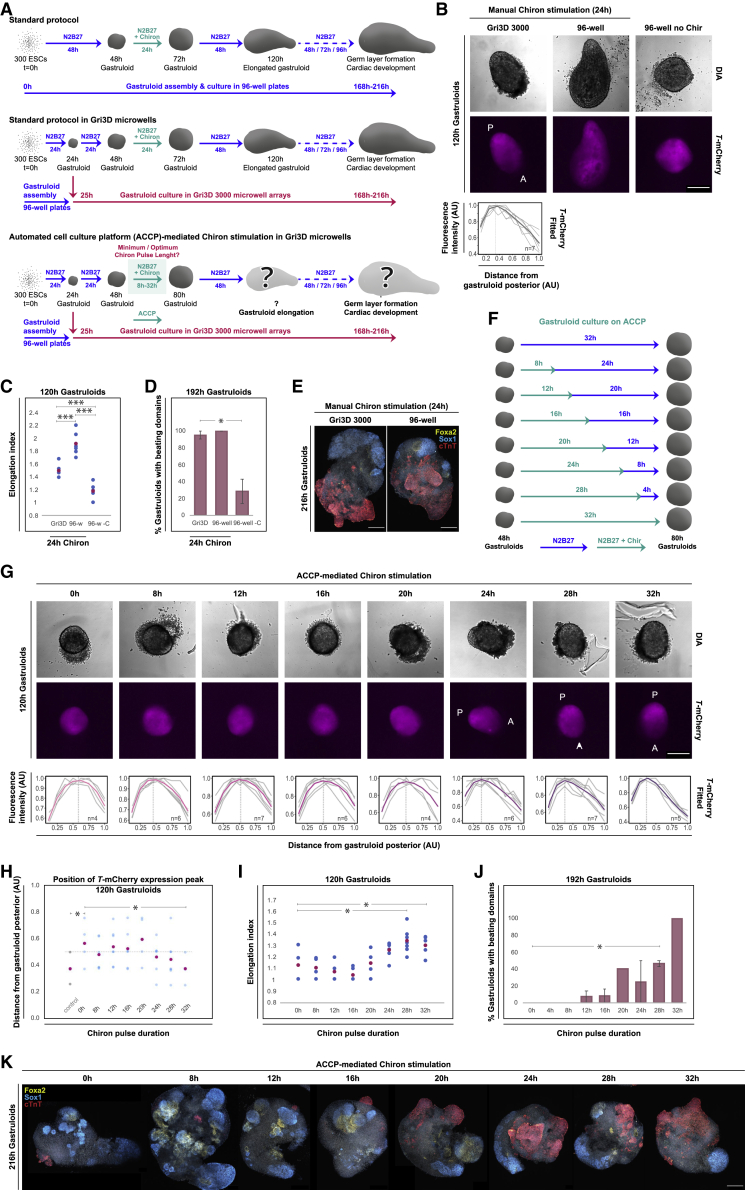
Developmental potential of 3D gastruloids in response to time-varying Chir stimulation (A) Overview of protocols used for the formation of mouse 3D gastruloids. Top: standard 96-well protocol. Middle: novel protocol for gastruloid cultures in Gri3D 3000 hydrogel microwell arrays. Bottom: protocol for ACCP-mediated Chir stimulation in Gri3D 3000 hydrogel microwell arrays. (B–E) Developmental potential of 3D gastruloids cultured in Gri3D microwell arrays versus gastruloid culture in conventional low-adhesion 96-well plates. (B) Characteristic images of 120-h gastruloids assembled from *Sox1*-GFP:*Brachyury*-mCherry (*SBr*) reporter ESCs with *T*-mCherry expression are depicted. A polynomial fit (bold line) through relative *T*-mCherry expression levels along the posterior-to-anterior axes of individual gastruloids (single lines) cultured in Gri3D microwells is shown. Scale bar, 250 μm. (C) Elongation index (length over width) of gastruloids cultured in the indicated conditions, with the mean colored in magenta. Gri3D, n = 7; 96-w, 96-well, n = 6; 96-w –C, 96-well without Chir stimulation, n = 7. ∗∗∗p ≤ 0.005 (unpaired one-tailed Student’s t test). (D) Percentage of gastruloids with beating structures at 192 h after aggregation. Averages from two independent biological experiments are presented. Error bars indicate ±SE. Gri3D, n = 21; 96-well, n = 21; 96-well –C, 96-well without Chir stimulation, n = 14. ∗p ≤ 0.05 (unpaired one-tailed Student’s t test). (E) Confocal images of 216-h gastruloids immunostained for the cardiac marker cTnT, the endoderm marker FOXA2, and the neuro-ectodermal marker SOX1. Scalebar, 250 μm. (F) Pulsing scheme for the automated Chir stimulation of 48-h gastruloids on the ACCP. (G–K) Developmental potential of *SBr* reporter gastruloids following time-varying Chir stimulation on the ACCP, cultured in Gri3D hydrogel microwell arrays. (G) Representative images of 120-h gastruloids, with polynomial fits (colored lines) through quantified *T*-mCherry expression distributions along the posterior-to-anterior poles of individual gastruloids, are shown. Scale bar, 250 μm. (H) Positions of the peaks of *T*-mCherry expression along the posterior-to-anterior axes of 120-h gastruloids. Mean values are indicated in magenta. N, numbers equal those indicated in (B) and (G). Control, gastruloids cultured in Gri3D microwells, with a manually administered 24-h Chir pulse. (I) Elongation of 120-h gastruloids. Mean values are depicted in magenta; 0 h, n = 4; 8 h, n = 6; 12 h, n = 6; 16 h, n = 6; 20 h, n = 4; 24 h, n = 6; 28 h, n = 7; 32 h, n = 5. ∗p ≤ 0.05 (unpaired one-tailed Student’s t test). (J) Percentage of 192-h gastruloids with beating domains. Graphs represent averages from duplicate (single for 4-, 20-, and 32-h Chir pulse lengths) biological experiments. 0 h, n = 12; 4 h, n = 7; 8 h, n = 13; 12 h, n = 14; 16 h, n = 12; 20 h, n = 5; 24 h, n = 10; 28 h, n = 13; 32 h, n = 5. Error bars denote ±SE. ∗p ≤ 0.05 (unpaired one-tailed Student’s t test). (K) Confocal images of 216-h gastruloids immunostained for cTnT, FOXA2, and SOX1. Scale bar, 250 μm. AU, arbitrary units; Chir, Chiron; –C, without Chiron stimulation.

First, we developed protocols for multiplexed gastruloid culture and exposure to Chir in Gri3D 3000 hydrogel microwell array 24-well plates (SUN bioscience; Figure 3A). Cellular aggregates are physically separated in individual microwells within the Gri3D hydrogel arrays, and they maintain their positions throughout medium exchange cycles (Brandenberg et al., 2020). With a medium reservoir adjacent to the aggregate-containing microwell arrays, Gri3D microwell plates are designed to enable near-complete liquid exchanges without aggregate loss during full medium replacement cycles (Figure S3). Integrating our system into Gri3D hydrogel microwell array plates thus supports the multiplexed culture and dynamic stimulation and the tracking of individual aggregates over time.

For multiplexed gastruloid culture in Gri3D microwell arrays, gastruloids were pre-formed through the aggregation of mouse *Sox1*-GFP:*Brachyury*-mCherry (*SBr*) reporter ESCs (Deluz et al., 2016) in low-attachment 96-well plates, using standard protocols (Baillie-Johnson et al., 2015; Rossi et al., 2021; Vianello et al., 2020a). Following 25 h of assembly and initial culture in 96-well format, single cellular aggregates were manually transferred into individual microwells of Gri3D hydrogel arrays (microwell diameter, 3,000 μm, with seven microwells per array). Gastruloids aggregated from *SBr* reporter ESCs and pre-formed in 96-well plates reproducibly (with an efficiency of 100%) elongated in Gri3D microwell arrays in response to a 24-h Chir stimulus, albeit at slightly slower timescales than when cultured under standard conditions in low-adhesion 96-well plates (Figures 3B, 3C, and S3). The gastruloids cultured in Gri3D microwells faithfully established an anterior-posterior axis, evidenced by the asymmetric expression of the early mesodermal marker *Brachyury* (*T*) 120 h after assembly. At 192 h, the majority of gastruloids had formed a beating compartment (Figures 3D and Video S2), which was further reflected in large domains staining positive for cardiac troponin T (cTnT) (Figure 3E), in line with previous observations of gastruloid cultures in standard 96-well conditions (Rossi et al., 2021).

Video S2. Time-lapse acquisition (DIA images) of a 192-h-old gastruloid, generated from *SBr* reporter ESCs and cultured in a Gri3D hydrogel microwell array, following a manually applied 24-h Chiron pulse (from 48 to 72 h after aggregate assembly), related to Figure 3 Time resolution, 500 ms. Scale bar, 250 μm.

Next, we examined developmental potential as a function of time-varying Wnt pathway activation. We applied Chir pulse lengths ranging from 8 to 32 h through the automated stimulation of 48-h-old gastruloids in Gri3D hydrogel microwell arrays on the ACCP, with medium exchange cycles every 4 h (Figures 3F, S3, and Video S3 as an example). A distinct, posterior *T*-mCherry pole first emerged in 120-h gastruloids in response to 24-h exposure to Chir. The posterior polarization of *T*-mCherry expression became more prominent with extended exposure times (Figures 3G, 3H, and S3). Correspondingly, a Chir pulse of 28 h or longer triggered robust elongation (Figures 3I and S3), and led to increased formation of beating foci in 192-h gastruloids compared with un-stimulated controls (Figure 3J, Data S1). Beating foci first emerged in a minor fraction of gastruloids following 12 h of Chir stimulation. These were observed at higher frequencies in gastruloids that had received Chir pulses of 20 h or longer and formed in all gastruloids examined in response to a 32-h Chir pulse. The gradual increase in the proportion of gastruloids that developed beating foci was accompanied by progressively larger cTnT-expressing domains, as a function of extended Chir pulse duration. It is conceivable that prolonged Chir stimulation (mimicking Wnt pathway activation) induces a lineage bias toward mesodermal derivatives, possibly at the expense of (neuro-)ectodermal (i.e., *Sox1*-positive) cell fate (Figure 3K).

Video S3. 32-h time-lapse acquisition (DIA images) of a 3D gastruloid, assembled from *SBr* reporter ESCs and cultured in a Gri3D hydrogel microwell array, during automated 24-h Chir stimulation on the ACCP, related to Figure 3 Time resolution, 4 h. Scale bar, 25 μm.

## Discussion

We present the design and development of a simple and low-cost, proof-of-concept DIY platform that enables fully automated, complex mammalian cell culture experiments under dynamically changing medium formulations. As a stand-alone platform, our DIY system is compatible with live-imaging, enabling real-time tracking of temporally varying medium formulations through supplemented fluorescent-dye tracers and direct observation of cellular and organismal behavior and cell fate outcomes.

We demonstrated the system’s advanced liquid handling and multiplexing capabilities through the generation of complex concentration profiles, offering users the unique ability to examine intricate stimulation-response relationships, and applied the ACCP to the investigation of two diverse stem-cell-based *in vitro* models for mammalian embryogenesis. Conducting automated medium switch experiments with high time resolution, we examined cellular commitment during the conversion of mouse naive ESCs into EpiLCs in 2D and the developmental potential of 3D gastruloids as a function of time-varying activation of the Wnt-signaling pathway through temporally modulated Chir stimulation.

ESCs proliferated and differentiated with known kinetics on the ACCP, highlighting the utility of our DIY system for the culture of sensitive primary cells. Performing fully automated, dynamic medium exchange experiments during the ESC-to-EpiLC transition, we refined the developmental point of no return along the developmental trajectory toward epiblast fate, at which the majority of cells have lost the ability to maintain an ESC-like state upon reversion into naive pluripotency-promoting conditions, previously suggested to occur between 24 and 48 h of culture in EpiLC-inducing conditions (Murakami et al., 2016), to lie around 29 to 35 h of EpiLC stimulation. Results from our proof of principle experiments, pointing to a likely sharp cell state transition and possibly “switch-like” behavior of the system, following an initial extended state of pluripotency, are highly congruent with findings on the timing of irreversible exit from naive pluripotency previously reported by Strawbridge et al. (Strawbridge et al., 2020). The exact molecular mechanism underlying a conceivable sudden and pronounced (switch-like) change in developmental potential upon exit from pluripotency warrants further investigation.

Next, by systematically varying the exposure duration of multiplexed 3D gastruloid cultures to the Wnt agonist Chir, we demonstrated a defined, time-dependent effect of Wnt pathway activation on early mammalian development. Concurrent with conventional protocols, a minimum Chir pulse of 24 h was required and sufficient to induce robust symmetry breaking, shown by the asymmetric expression of the mesodermal marker *Brachyury* (*T*) in gastruloids cultured for 120 h in Gri3D hydrogel microwell arrays and gastruloid elongation. Notably, the duration of Wnt pathway activation directly correlated with the formation of cardiac troponin T-expressing and beating domains. On the basis of the results from our time-resolved medium replacement experiments, we propose that extended Wnt pathway activation enhances the developmental potential for mesodermal derivatives, such as cardiac tissues, conceivably at the expense of (neuro-)ectodermal cell fates.

Through a series of proof-of-concept experiments, we have demonstrated the applicability of our fully automated DIY cell-culturing platform to ESC differentiation as well as emerging 3D *in vitro* cell culture models under dynamically changing medium compositions. Our system enabled the validation and further investigation of critical parameters underlying key developmental processes occurring during early mammalian development, which would have been cumbersome to achieve using conventional manual approaches.

The ACCP supports fully automated, multi-day cell culture experiments under dynamically changing medium formulations, desired in stem cell research, developmental biology, and toxicology studies. Time-resolved, automated medium-exchange experiments will be of immense value for providing deeper insight into cell fate commitment and cell fate specification programs more broadly (Gunne-Braden et al., 2020) and for identifying and establishing optimal and highly reproducible culture conditions. Notably, process automation and the development of precise cell culture systems will be of increasing importance both in academic settings and in industry, where robust protocols and well-controlled environments are critically important for good manufacturing practices (GMP) in cell and tissue engineering, biomedical applications, and regenerative medicine. The system’s potential to generate dynamically changing medium formulations further enables the delivery of complex conditions not readily achievable with conventional approaches. This capability facilitates, for example, systematic investigations on the functional consequence of signaling dynamics through time-varying modulations of pathway activities, such as the probing of defined frequencies and amplitudes, or the effect of the relative timing between signaling oscillations on cellular systems.

Our DIY platform is low cost and straightforward to build. The setup presented here includes PDMS microfluidic modules assembled by multi-layer soft lithography and cast from molds patterned with SU-8 and AZ photoresist. While the generation of molds using photolithography techniques requires access to clean-room facilities, which are available at most major research universities, custom molds can be obtained commercially (https://www.flowjem.com, https://www.su8masters.com) or can be 3D printed as an alternative to photolithography (de Almeida Monteiro Melo Ferraz et al., 2020; van Oostrom et al., 2021). PDMS device fabrication can be performed in a non-specialized laboratory setting, requiring minimal equipment such as a spin-coater, stereomicroscope, and oven. The microfluidic modules are controlled by standard solenoid valves and a USB relay board, which are commercially sourced (Brower et al., 2018; White and Streets, 2018; Wong et al., 2018). Alternatively, millifluidic systems as recently described (Wong et al., 2018) could be used to substitute for the microfluidic modules described here.

Mimicking standard tissue culture techniques, such as gentle medium addition, and removal via aspiration through medium efflux tubing directed toward the edge of the culture chamber, the ACCP circumvents shear-induced effects, frequently observed in microfluidic cell culture systems, and minimizes cell detachment and loss during automated medium exchange cycles.

By integrating with conventional multi-well tissue culture plates, our system offers considerable flexibility and thus compatibility with current and future *in vitro* cell culture models in 2D and 3D. Combining our platform with standard 24-well polystyrene plates (nunc, Life Technologies), imaging-compatible 24-well plates (ibidi u-plates), and Gri3D hydrogel microwell arrays (SUN bioscience), we showcased the adaptability and versatility of the ACCP and demonstrated its applicability and value for the dynamic culture and stimulation of mouse ESCs and 3D gastruloids. Straightforward integration into microwell array plates renders our system compatible with the multiplexed culture of cellular aggregates (i.e., embryoids, gastruloids, organoids), and single-aggregate tracking (Brandenberg et al., 2020).

The system’s DIY nature and modular design facilitates the straightforward re-configuration to particular experimental requirements, such as the type and dimensions of the culture wells, culture volume, and full versus partial medium removal during exchange cycles, while permitting standard downstream cellular and molecular analyses. For example, one might wish to integrate our system with AggreWell plates (STEMCELL Technologies), which are widely used for the culture of embryoids and organoids. AggreWell plates are highly similar in concept and design to the Gri3D hydrogel microwell arrays (SUN bioscience) that we employed for the multiplexed culture and time-varying Chiron stimulation of gastruloids. However, whereas Gri3D hydrogel microwell arrays are specifically designed with a medium reservoir adjacent to the aggregate-containing microwell arrays, thereby enabling near-complete medium exchanges without aspirating or displacing cellular aggregates, fluid exchange lids for AggreWell plates would need to be configured to facilitate fractional medium exchange cycles. Partial medium exchanges can straightforwardly be accomplished by adjusting the length of the efflux tubing reaching into the culture wells such that they end slightly above the microwells. Thus, as a simple-to-implement alternative to performing full medium exchanges with every automated cycle, fractional medium exchanges might be valuable for the automated culture and dynamic stimulation of cellular aggregates in AggreWell (or similar) plate formats, where complete medium exchange cycles might not be desired or beneficial. We further envision adaptation of the platform for the delivery of dynamic inputs to a variety of widely employed model systems *in vivo* and *ex vivo*, such as mouse and zebrafish embryos, cultures of tissue explants, and patient-specific organoids.

We have demonstrated integration of our system with concurrent and downstream applications, such as time-lapse microscopy in conjunction with quantitative image analysis, phenotypic characterization, flow cytometry, and immunohistochemistry. Our DIY platform can, in principle, be combined with other cell- and molecular-biology techniques of interest, including (single-cell) RNA-seq, qRT-PCR, ChIP-seq, and metabolic profiling, for the in-depth characterization of the acquired cellular states or developmental stages.

One could also imagine integration of additional functionalities into the ACCP, such as pre-programmed cell fixation and subsequent wash steps for the automated capturing of cellular or developmental states at specific points in time. This capability could be directly coupled with automatically performed downstream molecular-biology assays for characterization.

The current design and setup of the ACCP enables addressing eight parallel culture wells with individual medium formulations and stimulation dynamics. Increasing the multiplexing capability to 16–24 culture wells can be accomplished by drilling additional access ports into the plate lid, fitting tubing for additional fluidic interconnects, and expanding the multiplexing modules, pneumatic setup, and the software (LabVIEW code) controlling fluidic operations. Of note, the time resolution between medium exchange cycles will decrease with increased system throughput. Full medium exchange cycles in all eight parallel culture wells could be accomplished within an hour. In most cellular systems, and during vertebrate embryonic development, a time resolution of 1–3 h is largely sufficient to enable the systematic investigation of complex dynamic phenomena. Of course, an alternative and even simpler approach to scaling is by simply duplicating the entire ACCP.

Our proof-of-concept ACCP system enables researchers to perform complex cell culture experiments under fully automated, dynamically changing medium formulations using existing standard multi-well culture plates and protocols. We anticipate that the ACCP will provide a foundation for more extensive analysis and control of cellular behavior, and, because of its DIY nature, it can be readily adopted by the broader scientific community.

### Limitations of the study

With the engineering efforts and validation studies presented in this report, we aimed to provide a powerful, yet simple-to-implement, proof-of-concept technology for the automated culture, dynamic stimulation, and direct observation of mammalian cells, in particular of emerging physiologically and medically highly relevant, complex 3D cell culture systems. We recognize that, in its current version, our proof of principle system is not operating at high throughput and that, within the scope of this paper, we have not yet showcased the full potential of the ACCP in terms of applying complex medium formulations to relevant biological systems.

At present, our proof-of-concept system enables addressing eight parallel culture chambers with individual, time-varying medium compositions. We demonstrated several of the advanced capabilities and key advantages of our system, such as the generation of complex concentration profiles through fluorescent dye mixing and concurrent time-lapse microscopy, and automated medium exchanges at high time resolution during EpiLC stimulation and 3D gastruloid culture. We acknowledge that it might, in principle, be possible to perform some of the featured medium exchange routines manually. Notably, however, our automated cell-culturing experiments presented here were performed over timescales of 32–48 h, with medium-exchange cycles every 1–6 h. We consider manual frequent medium exchanges over such extended periods to be cumbersome, error prone, and generally impractical. Furthermore, recurrent manual manipulations in conjunction with time-lapse imaging are problematic. Moving cell culture plates back and forth between the microscope stage and a sterile flow hood poses a challenge for maintaining exact imaging positions, while recurring medium exchanges performed directly on the microscope stage increase the risk of contamination.

We further demonstrated integration of our system with standard downstream cell- and molecular-biology applications and performed basic characterizations of the acquired cellular and developmental states. Performing proof-of-concept experiments, we validated previous findings on the timing and exit from naive pluripotency, established novel paradigms, and generated additional testable hypotheses. We acknowledge that within the scope of this report, with a focus on method development, the cell-biological characterizations presented here are not exhaustive. Additional endpoint measurements, such as, for example, expression analysis of the primed pluripotency marker OTX2 through immune-staining or qRT-PCR profiling, following EpiLC stimulation by bFGF and activin A, would complement investigations on the loss of naive pluripotency, assessed through quantitative analyses of *Rex1*-GFP expression, and further confirm ESC-to-EpiLC conversion.

Comparing cellular proliferation rates, reporter gene expression levels, and spatial expression distributions of parallel cultures of mouse ESCs induced to convert into EpiLCs and 3D gastruloids, grown under conventional batch culture conditions versus under frequent medium exchanges on the ACCP, we observed no obvious compromising effects on cellular behavior and cell fate specification upon high-frequency medium exchange cycles. However, we recognize that recurrent medium exchanges might pose a challenge for the culture of cells in sub-optimal conditions where cells rely extensively on the secretion of—potentially undefined—endogenous factors. As such, we acknowledge that certain cell types or differentiation protocols might not be compatible with frequent medium replacements, and, consequently, the investigation of complex stimulation profiles. However, although the ACCP might not provide a valuable option for every cell-culturing protocol, we wish to emphasize that our platform can be easily adapted for culture systems that are more reliant on endogenous secreted factors; instead of performing complete medium exchanges with every cycle, increasing the distance of the efflux tubing from the bottom of the culture chamber readily enables user-defined, partial medium exchanges, thereby reducing wash-out of endogenous cell-secreted factors that might be required to support robust stem cell self-renewal or differentiation into specific cell fates.

In conclusion, enabling fully automated, multi-day cell culture experiments under temporally modulated medium compositions, we envisage that our prototype DIY system will open up new avenues of investigation in stem cell research, developmental biology, and regenerative medicine.

## STAR★Methods

### Key resources table

**Table:** 

REAGENT or RESOURCE	SOURCE	IDENTIFIER
**Antibodies**		

Rabbit monoclonal anti-FOXA2	Abcam	Cat# ab108422; RRID: AB_11157157
Goat polyclonal anti-SOX1	R&D Systems	Cat# AF3369; RRID: AB_2239879
Mouse monoclonal anti-Cardiac Troponin T (cTnT)	Thermo Fisher Scientific	Cat# MA5-12960; RRID: AB_11000742

**Chemicals, peptides, and recombinant proteins**		

StemMACS CHIR99021 in Solution	Miltenyi Biotec	Cat# 130-106-539
StemMACS PD0325901 in Solution	Miltenyi Biotec	Cat# 130-106-541
CHIR99021 (3D gastruloid stimulation)	Merck/Millipore	Cat# 361559
Recombinant Human/Murine/Rat Activin A	PeproTech	Cat# 120-14-10
Recombinant Human bFGF	Thermo Fisher Scientific	Cat# 13256029
Alexa Fluor 647-Dextran, 10,000 MW	Thermo Fisher Scientific	Cat# D22914
Fluoresceinisothiocyanat (FITC)-Dextran, 10,000 MW	Sigma	Cat# FD10S
CellTrace Violet Cell Proliferation Kit	Thermo Fisher Scientific	Cat# C34557

**Experimental models: Cell lines**		

*Rex1*-GFP Mouse ESCs	Laboratory of Austin Smith	E14Tg2a; RRID: CVCL_9108
*Sox1*-GFP::*Brachyury*-mCherry (*SBr*) Mouse ESCs	Laboratory of David Suter	CGR8, strain 129; RRID: CVCL_3987
*SBr* WT Mouse ESCs; Parental strain corresponding to *Sox1*-GFP::*Brachyury*-mCherry (*SBr*)	Laboratory of David Suter	CGR8, strain 129; RRID: CVCL_3987

**Software and algorithms**		

AutoCAD	Autodesk	https://www.autodesk.com
Fiji/ImageJ	Schindelin et al., 2012	RRID: SCR_002285; https://imagej.net/software/fiji/
FlowJo	BD	RRID: SCR_008520; https://www.flowjo.com
LabVIEW v14.0	National Instruments	RRID: SCR_014325; https://www.ni.com/en-us/shop/labview.html
NIS-Elements	Nikon	RRID: SCR_014329; https://www.nikonmetrology.com/en-us/industrial-microscopes/nis-software-nis-elements-microscope-imaging-software
LabVIEW scripts for microfluidic device operation	This paper, deposited on Zenodo	https://doi.org/10.5281/zenodo.6579452
Fiji script to calculate gastruloid elongation indices	Guiet et al., 2021	https://doi.org/10.5281/zenodo.4544369
Custom processing pipeline for quantifying fluorescent intensities (reporter gene expression levels) along gastruloid posterior-to-anterior poles	This paper, deposited on Zenodo	https://doi.org/10.5281/zenodo.5717752

**Other**		

Design files (AutoCAD) for microfluidic devices	This paper, deposited on Zenodo	https://doi.org/10.5281/zenodo.6579452

### Resource availability

#### Lead contact

Further information and requests for resources and reagents should be directed to and will be fulfilled by the lead contact, Sebastian J Maerkl (sebastian.maerkl@epfl.ch).

#### Materials availability

This study did not generate new unique reagents.

### Experimental model and subject details

#### Cell lines

*Rex1*-GFP (Kalkan et al., 2017; Wray et al., 2011), *Sox1*-GFP::*Brachyury*-mCherry (*SBr*) (Deluz et al., 2016) reporter mouse embryonic stem cells (ESCs) and corresponding wild type (*SBr* WT) mouse ESCs were used in this study. Naive *Rex1*-GFP and *SBr* WT mouse ESCs were maintained at 37°C, in a 5% CO_2_ atmosphere, in N2Diff 227 (Takara, Y40002) supplemented with 1μM PD0325901 (Miltenyi Biotec, 130-106-541), 3μM CHIR99021 (Miltenyi Biotec, 130-106-539), 100 ng mL^−1^ LIF (Protein Facility, EPFL, Lausanne) (‘2i/LIF’-media (Ying et al., 2008)), and 100U ml^−1^ Penicillin-Streptomycin (PS; Life Technologies, 15140122) on 0.1% gelatine-coated (Fluka, 48723) multi-well cell culture dishes (Falcon, 353043 and 353046) or flasks (TPP, 90026). Cells were passaged every 2–3 days, using Accutase (Life Technologies, A1110501) for gentle dissociation (2–5 minutes) at room temperature. Media was exchanged on alternate days.

### Method details

#### Induction of epiblast-like cell fates

*Rex1*-GFP and *SBr* WT mouse ESCs were employed as an experimental model system for the ESC-to-epiblast-like cell (EpiLC) transition (Hayashi et al., 2011).

The day before EpiLC induction, media was replaced with 2i/LIF-media supplemented with knockout serum replacement (KSR; Life Technologies, 10828010) to a final concentration of 1%. For EpiLC stimulation, approximately 25.000 ESCs were seeded per cm^2^ of culture dishes, pre-coated over-night with 16.67ug mL^−1^ human plasma fibronectin (Millipore, FC010), in N2Diff 227 (Takara, Y40002) supplemented with 20 ng mL^−1^ activin A (PeproTech, 120-14-10), 12 ng mL^−1^ bFGF (Life Technologies, 13256029), 1% KSR (Hayashi et al., 2011), and 100U mL^−1^ PS. Media was exchanged once (after around 24h) during the 48h (±1h) ESC-to-EpiLC conversion.

#### 3D gastruloid culture

3D gastruloids were generated from *Sox1*-GFP:*Brachyury*-mCherry (*SBr*) reporter ESCs. For maintenance, naive *SBr* reporter ESCs were cultured in DMEM (Gibco/Life Technologies, 61965-059) supplemented with 10% ESC-grade bovine fetal calf serum (FCS; Gibco/Life Technologies, 16141-079), 1μM PD0325901 (Miltenyi Biotec, 130-106-541), 3μM CHIR99021 (Miltenyi Biotec, 130-106-539), 100 ng mL^−1^ LIF (Protein Facility, EPFL, Lausanne), and 100U mL^−1^ PS on multi-well tissue culture dishes (Falcon, 353046) or flasks (TPP, 90026), without pre-coating. Cells were passaged every 2–3 days, through gentle dissociation with Accutase (Life Technologies, A1110501), for 2–5 minutes at room temperature. Media was replaced on alternate days.

For gastruloid assembly and culture, a standard protocol for gastruloid formation (Baillie-Johnson et al., 2015; Rossi et al., 2021; Vianello et al., 2020a) was employed. Briefly, approximately 300 ESCs were aggregated in 40μL N2B27 supplemented with 100U mL^−1^ PS (N2B27/PS) in individual wells of 96-well Clear Round Bottom Ultra-Low Attachment Microplates (Corning, 7007). At 48h after aggregation, 150μL of 3μM CHIR99021 (Chir; Merck/Millipore, 361559) in N2B27/PS were added to each well. Media was replaced with 150μL N2B27/PS at 72h following gastruloid assembly, and in 24h-intervals up to a total of 168h after gastruloid formation.

For multiplexed gastruloid culture in Gri3D hydrogel microwell arrays, single gastruloids assembled in 96-well Clear Round Bottom Ultra-Low Attachment Microplates (Corning, 7007) (standard protocol) were transferred in 20μL N2B27/PS into individual microwells of pre-conditioned (N2B27/PS, over-night) Gri3D hydrogel arrays (24-well plate format, microwell diameter, 3000μm, with seven microwells per array; SUN bioscience, Gri3D-24P-L-8) by 25h of culture. Array wells were topped up with 860μL of N2B27/PS for a final culture volume of 1000μL. 48h after gastruloid assembly, 850ul N2B27/PS were removed from the array wells, and gastruloid cultures were stimulated with 1050μL of 3μM CHIR99021 (Merck/Millipore, 361559) in N2B27/PS. To keep media ratios in the microwell arrays consistent with standard gastruloid culture conditions in 96-well culture plates, further 130ul of N2B27/PS were added per array well, for a final culture volume of 1330μL (a culture volume of 150ul remains within the hydrogel microwell array). Following 72h of aggregation, media in the array wells was replaced with N2B27/PS (1180ul per well). For continued gastruloid culture in Gri3D microwell arrays, N2B27/PS was replaced at daily intervals, up to 168h after the initial gastruloid assembly. Using this approach, gastruloids pre-formed in 96-well plates developed into elongated structures upon transfer and continued culture in Gri3D hydrogel microwell (3000μm diameter) arrays, with an efficiency of 100%, standard protocols alike.

The handling of Gri3D hydrogel microwell array plates, such as the transfer of plates from the laminar flow hood to the incubator, microscope for visual inspection, or microfluidic setup, may lead to the occasional floating of individual gastruloids out of their microwell and into an adjacent microwell, or into the media reservoir. This likely is a common feature of microwell-plates. As gastruloids tend to fuse into larger structures when placed into close proximity, we excluded wells that had acquired two gastruloids per microwell from *T*-mCherry expression distribution and elongation analyses. Following this "quality control step," and discounting empty wells, our numbers of gastruloids ranged between four to seven (out of a maximum of seven) gastruloids per 24-well microwell array.

#### Cell proliferation assay

To quantitatively assess cellular proliferation, ESCs were stained with CellTrace Violet Cell Proliferation Kit (Molecular Probes, Life Technologies, C34557) at a final concentration of 2.5μM, according to manufacturer’s instructions for the labeling of adherent cells. CellTrace Violet dye levels were quantified by flow cytometry following 48h of EpiLC stimulation, and in freshly labeled ESCs.

#### Flow cytometry

For flow cytometry analysis, cells were harvested and resuspended in 1xPBS (Gibco/Life Technologies, 20012-027) supplemented with BSA (Gibco/Life Technologies, 15260-037) to a final concentration of 3%. Cells were stained with 5 μg mL^−1^ propidium iodide (PI, Molecular Probes, Life Technologies, P3566) to enable discrimination between live and dead (PI-positive) cells. Flow cytometry-based quantification of fluorescence levels was performed on a BD LSR II SORP analyzer. Data were evaluated using FlowJo software.

#### Immunohistochemistry

Gastruloids were fixed over-night at 4°C in a 4% paraformaldehyde in PBS solution (Thermo Scientific, 15434389). Immune-labelling of gastruloids was performed as described previously (, Vianello et al., 2020b). Primary antibodies used were as follows: rabbit anti-FOXA2 (Abcam, ab108422; 1:500), goat anti-SOX1 (R&D Systems, AF3369; 1:200), and mouse anti-cTnT (Invitrogen/Thermo Fisher Scientific, MA5-12960; 1:50). Nuclei were stained with 2 μg mL^−1^ DAPI (Invitrogen/Thermo Fisher Scientific, D1306). Confocal Images were acquired on a Leica SP8 UP2 microscope. Images were processed using Fiji software (Schindelin et al., 2012). Fluorescent image intensity scales were adjusted equally.

#### DIY system for automated cell culture under dynamically changing media formulations

##### Microfluidic device fabrication

The microfluidic control modules are low-complexity, simple-to-fabricate, two-layer polydimethylsiloxane (PDMS) devices (Thorsen et al., 2002). Molds for microfluidic modules were fabricated using standard photolithography methods. Flow channels were patterned to a height of 40μm in AZ 40XT (Micro-Chemicals) positive photoresist, control layer channels were patterned in SU-8 GM1070 (Gersteltec) negative photoresist to a height of 30μm. Microfluidic modules were cast in PDMS (Sylgard 184 Silicone Elastomer Kit, Dow Corning Corp., USA) and assembled through multi-layer soft lithography methods (Thorsen et al., 2002). Devices were bonded to glass microscope slides (VWR, 631–1550) following 20s of oxygen plasma treatment, and baked at 80°C for 2h to over-night. Microfluidic devices were designed in AutoCAD software. Design files are available at https://doi.org/10.5281/zenodo.6579452.

##### Characterizing the microfluidic PWM module

The microfluidic pulse width modulation (PWM) media formulator is a two-layer PDMS device, with six media inflow channels of equal length converging into one flow mixing channel as previously described (Woodruff and Maerkl, 2018). Key to producing desired concentrations with high precision is the diffusion of PWM-generated, alternating input pulses to homogeneous output solutions. Mixing of individual pulses to homogeneity is a function of the path length between the microfluidic PWM module and media dispensing in the culture wells, the amount of time occupied by a single pulse (duty cycle time), and the flow rate. We determined optimal operational parameters through evaluating the mixing to homogeneity of a 7.5μM fluoresceinisothiocyanat (FITC)-dextran 10 kDa solution (Sigma, FD10S) and buffer (3% BSA [Gibco/Life Technologies, 15260-037] in MilliQ water), via microfluidic PWM at increasing fluidic path lengths and cycle times (the sum of the two alternating liquid pulses), at an inflow pressure of 10 psi (∼69kPa). Output solutions exiting a single outlet of the microfluidic PWM and multiplexing (PWM-MUX) module were routed via flexible PTFE tubing (Adtech Polymer Engineering/Fisher Scientific, 11929445, with an inner diameter of 0.56 mm) into a microfluidic flow channel, where fluorescent images were acquired with a time-resolution of 500ms. A fluidic path length of 37.5 cm (PTFE tubing, with an inner diameter of 0.56 mm) resulted in homogeneous outflow solutions at a maximum inflow pressure of 10 psi and cycle time of 1.5s, with individual input pulses (duty cycles) ranging from 0.1 to 0.9 of the total cycle times (Figure S1). All experiments that involved liquid routing through the PWM-MUX module were performed at an inflow pressure of 10 psi (∼69kPa), and a total cycle time of 1.5s, with a minimum programmed duty cycle of 100ms.

#### Implementing half-wash cycles to clear the microfluidic device and inflow tubing from preceding media formulations

In order to prevent media formulations from preceding PWM cycles, remaining as "dead volume" within the PWM-MUX module and connecting tubing, from contaminating the media compositions formulated by the active PWM cycle, half-wash cycles were integrated within the dynamic operation mode of the automated cell culture platform (ACCP). Half-wash cycles were achieved through the topping up of the culture well to 150% of its volume with the newly generated media formulation, followed by complete emptying, re-filling to its standard volume, and re-setting of the liquid level.

#### Engineering the fluidic control lid

Re-usable fluid exchange lids were assembled for custom 24-well polystyrene cell culture plates: nunc (Life Technologies, 142,475), ibidi u-plates (ibidi, 82,426), and Gri3D hydrogel microwell arrays (SUN bioscience, Gri3D-24P-L-8). Access ports for three (four, for in-well CO_2_ perfusion for gastruloid cultures) interconnects per individual culture well were drilled into the plate lid using a standard hand-held electric drill. A thin layer (approximately 30 mL) of PDMS (Sylgard 184 Silicone Elastomer Kit, Dow Corning Corp., USA), at a monomer to catalyst ratio of 10:1, was cast into the lid and cured at room temperature for 2 days. The PDMS layer was pierced, and PTFE tubing (Adtech Polymer Engineering/Fisher Scientific, 11929445), at a length of 37.5 cm for media inflow, and 25 cm for media leveling, emptying outflow, and optional in-well CO_2_ perfusion was inserted through the pierced plate lid, entering individual culture chambers through the drilled access ports from the top. For additional stability, and to facilitate straight-forward modifications of fluidic interconnects reaching into the culture wells, such as varying the insertion depth in order to readily re-adjust the culture volume, small metal tubing was inserted into the end of each PTFE tube. The metal tubing was obtained from dispensing needles with luer stub adapters (Metcal, 923050-TE and 923,050-45BTE). Media efflux tubing was fitted with a kinked metal pin, directed at the edge of the well, to prevent aspiration of cells during full emptying cycles.

The final culture volume, and the volume of medium that is replaced during each liquid exchange cycle, is set by the distance of the metal pins from the bottom of the culture well. Tubing length and insertion depth, type, and shape of the metal pins can be tailored to specific experimental parameters and requirements. For the experiments presented, fluidic control lids were configured to a final culture volume of approximately 750μL per standard 24-well (nunc, ibidi u-plates), and about 1330μL for the Gri3D hydrogel microwell arrays, with near-complete liquid removal accomplished through tubing reaching to the bottom of the culture well. We note that for the culture of gastruloids in Gri3D hydrogel microwell array plates, full (as-complete-as-possible) media removal leaves a culture volume of 150ul within the Gri3D 3000 microwell arrays. This residual amount is defined by the design of the Gri3D microwell arrays, and is required in order to prevent loss of cellular aggregates floating within individual microwells.

#### Operating the ACCP

Prior to assembling the ACCP on the motorized stage of a Ti-E Eclipse microscope enclosed in a temperature control chamber (Nikon), set to 37°C, microfluidic modules, media inflow and outflow collection bottles, with attached tubings, were autoclaved. Fluidic interconnects of the re-usable fluidic control lid were flushed with 70% ethanol, and the lid was placed under UV in a sterile laminar flow hood, with media-interfacing tubing and metal pins facing up. The standard lid of a corresponding, sterile multi-well tissue-culture plate was replaced with the ethanol-cleaned and UV-treated, engineered plate lid, and tubings of the fluid exchange lid, fitted with small metal tubing (Unimed, AISI 304, 0.65/0.35 × 8 mm), were connected to the microfluidic modules: fluidic interconnects for media inflow into the individual cell culture chambers were inserted into the outlets of the microfluidic PWM-MUX module, media outflow tubings (8 each for level setting and emptying) were routed into the inlets of single microfluidic channels. The outlets of the microfluidic channels controlling media outflow were individually connected to two liquid collection bottles (one each for collecting leveling and emptying outflow), connected to a vacuum pump (Figures 1A and 1C). For the culture of 3D gastruloids on the ACCP, additional interconnects for direct CO_2_ perfusion into individual wells were connected to two five-way manifolds with luer lock connectors, which were connected to a gas-bottle of pre-mixed 5% CO_2_ (Figure S1). The 5% CO_2_ was routed through a home-made CO_2_ humidifier prior to being distributed by the five-way manifolds to the multi-well plates.

Microfluidic control layers were pressurized using a custom pneumatic setup. Control lines for the microfluidic inflow (PWM-MUX) module were primed with MilliQ water. Control valves for the microfluidic outflow modules were air-pressurized, and operated through alternating pressure (for closure) and suction (for re-opening, via an attached vacuum pump).

Media inflow was pressure-driven and could be easily adjusted by tuning the pressure. For media supply, tubings from pressurized bottles containing media (Figure S1) were plugged into inlets of the PWM-MUX module. Pressurizing media-containing bottles with pre-mixed 5% CO_2_ saturated the media and supported culturing of CO_2_-dependent cells. Media was kept in reservoirs under CO_2_ pressure, at 37°C, for a maximum of 48h. This corresponds to standard culturing conditions in incubators, where naive embryonic stem cells are maintained at 37°C under CO_2_ atmosphere, routinely with media exchanges on alternate days.

Initially, we empirically determined the times required to empty, re-fill, and re-set the culture volume of individual wells. These measured parameters, and the desired frequency of media exchange cycles were specified in the software’s graphical user interface for fully automated operation (LabVIEW). LabVIEW scripts can be easily modified to program custom concentration profiles. Experimental parameters used for programmed liquid exchanges on the ACCP are summarized below in Table, Summary of programmed settings for experiments performed on the ACCP.

Once all parameters were established, cells pre-seeded (for 3-5h for ESC-to-EpiLC induction experiments, and for 48h for 3D gastruloid stimulation) in the corresponding multi-well plates were placed under the fluidic control lid. Imaging positions were selected, and fully automated media selection and exchange cycles, and time-lapse acquisition (NIS, Nikon) were started.

For timed media switch experiments during the ESC-to-EpiLC transition, cells were seeded into EpiLC-inducing medium (N2Diff supplemented with bFGF and activin A) and grown in batch culture in a standard tissue culture incubator for an initial 5h of EpiLC stimulation, in order to allow cells to settle down and attach to the bottom of the culture wells, prior to 42h of culture on the ACCP, with automated media replacement cycles. For time-varying Chir pulse experiments, 48h old 3D gastruloids were cultured on the ACCP for 32h. Subsequent media exchanges were performed manually, at daily intervals.

In order to enable tracking of the respective media formulations during timed media exchange experiments performed on the ACCP, 2i/LIF media was spiked with Alexa Fluor 647-dextran (10 kDa; Invitrogen, D22914; 0.25μM final concentration), and FITC-dextran (10 kDa) was added to N2B27 plus Chir at a concentration of 2.5μM.

To maintain sterility in the system, all solutions were supplemented with 100U ml^−1^ PS.

**Table:** Summary of programmed settings for experiments performed on the ACCP

	*EpiLC induction 1-plex*	*Complex patterns & EpiLC induction/reversion 8-plex (PWM-MUX)*	*3D gastruloid stimulation 8-plex (PWM-MUX)*
***Plate format***	24-well (nunc)	24-well (ibidi)	24-well Gri3D 3000 (SUN biosciences)
***Set culture volume (μL)***	750	750	1330
***Inflow pressure [psi]***	5	10	10
***Emptying time [s]***	45	45–120	90
***Filling time [s]***	75	150–180	240–270
***Leveling time [s]***	30	40–60	60

Emptying, filling, and leveling times refer to the times required to fully empty and re-fill one culture well, and re-set the culture volume to its pre-defined volume, as set in the LabVIEW user interface.

### Quantification and statistical analysis

#### Image acquisition and quantitative analysis

Imaging during ACCP operation and thereafter was performed on a Ti-E Eclipse automated microscope equipped with a temperature control chamber, and NIS-Elements software (Nikon). 14-bit images were acquired with an Andor DU-888 camera and processed using Fiji software (Schindelin et al., 2012).

For quantitative analysis, image correction was performed. The average intensity projection of 100 acquired darkfield images was subtracted from raw fluorescent images, before normalizing the darkfield-corrected images by the darkfield-subtracted, median intensity projection of 100 flatfield images, acquired at different positions. Median fluorescence intensities of darkfield- and flatfield-corrected images were quantified within an area of 100 × 100 pixels.

To account for potential small differences in the distances between the bottom of the culture wells and the level setting tubings/pins, which determine the liquid volume in individual culture wells, and thus directly relate to fluorescence intensity measurements, ACCP-generated, darkfield- and flatfield-corrected median fluorescence intensity values were normalized by median fluorescence intensities measured for 0.5μM FITC-dextran 10kDa solutions in the corresponding wells (generation of complex concentration profiles, Figures 1G–1J and S1). A 0.5μM FITC-dextran 10kDa solution corresponds to the lowest concentration generated in all experiments through the automated dynamic on-chip mixing of a 7.5μM FITC-dextran 10kDa solution and buffer.

In order to transform relative fluorescence intensity measurements into concentration values, a series of fluorescence intensity measurements of pre-mixed FITC-dextran 10kDa solutions of known concentrations were performed, and linear fits were established (concentration curves, Figure S1). Parameters derived from the underlying linear fits were used to calculate absolute concentrations from measured median fluorescence intensity values, following darkfield- and flatfield-correction, and normalization.

To aid visualization of displayed brightfield (DIA) images and time-lapse movies of ESCs/EpiLCs and 3D gastruloids, brightness and contrast were individually adjusted.

#### Calculating the elongation index of gastruloids

To quantify elongation of gastruloids by 120h after aggregation, a custom-written Fiji plugin (Guiet et al., 2021) was employed, with adaptation, to derive elongation indexes from darkfield-and flatfield corrected fluorescent (*T*-mCherry) images, following thresholding using maximum entropy (‘MaxEntropy’). Elongation indexes are calculated as the length of the gastruloid divided by the diameter of the largest circle that could be fit within the width of the gastruloid.

#### Quantification of polarized *Brachyury* (*T*) expression in gastruloids

In order to measure *T*-mCherry reporter expression levels along the posterior-to-anterior axis, a custom processing pipeline (Vianello et al., 2020c) was adapted to quantify signal intensities from darkfield- and flatfield-corrected fluorescent images of 120h gastruloids. A montage of all individual gastruloid images acquired for a given condition (specific Chir pulse length) with manually defined central axes of gastruloids, was provided as input. Gastruloids were computationally divided into ten segments of equal length, and total fluorescence intensities across a width of 80 pixels were computed for each segment, assigning signal intensities to defined positions along the posterior-to-anterior axes. For each gastruloid, extracted raw fluorescence values were normalized by the maximum fluorescence intensity value measured in the individual gastruloid, and to its length.

To enable comparison of *T*-mCherry expression patterns between conditions (varying Chir pulse lengths), polynomial functions were fit to normalized fluorescence signal intensities distributed along the posterior-to-anterior axes of gastruloids cultured under any given condition (n = four to seven gastruloids per individual condition). Residuals (difference between the fluorescence intensity measured and that of the polynomial fit at any given position) were also calculated. For comparative analyses across conditions, statistics were calculated by taking in account the axial position corresponding to the maximal fluorescence expression value recorded in each individual gastruloid. The mean of all such values was taken as the summary statistics for each given condition.

#### Statistical analysis

Microsoft excel was used for statistical evaluation of data. Unpaired (heteroscedastic) 1-tailed Student’s *t*-tests were employed for determining statistical significances. Statistical details, such as the number of independent biological experiments, sample size, definition of center, dispersion, and significance are described in the figure legends. Significance levels are expressed as follows: ∗, p ≤ 0.05; ∗∗∗, p ≤ 0.005.

## Data Availability

• All data reported in this paper will be shared by the lead contact upon request. • All original code is available at https://doi.org/10.5281/zenodo.6579452 (LabVIEW scripts for operating microfluidic devices) and https://doi.org/10.5281/zenodo.5717752 (custom processing pipeline for quantifying fluorescent intensities (i.e., *T*-mCherry expression distribution) along the gastruloid posterior-to-anterior poles). The DOIs are also listed in the Key resources table. • Any additional information required to reanalyze the data reported in this paper is available from the Lead contact upon request.

## Supplemental information

Document S1. Figures S1–S3

Data S1. Movies showing time-lapse acquisitions (DIA images) of 192-h-old gastruloids, generated from *SBr* reporter ESCs and cultured in Gri3D hydrogel microwell arrays, following time-varying Chiron stimulation (20, 24, 28, 32 h) on the ACCP (from 48 h after aggregate assembly), related to Figure 3 Time resolution, 500 ms. Scale bar, 250 μm.

Document S2. Article plus supplemental information

## References

[bib1] Ainla A., Gözen I., Orwar O., Jesorka A. (2009). A microfluidic diluter based on pulse width flow modulation. Anal. Chem..

[bib2] Azizi F., Mastrangelo C.H. (2008). Generation of dynamic chemical signals with pulse code modulators. Lab Chip.

[bib3] Baillie-Johnson P., van den Brink S.C., Balayo T., Turner D.A., Martinez Arias A. (2015). Generation of aggregates of mouse embryonic stem cells that show symmetry breaking, polarization and emergent collective behaviour in vitro. JoVE.

[bib4] Beccari L., Moris N., Girgin M., Turner D.A., Baillie-Johnson P., Cossy A.-C., Lutolf M.P., Duboule D., Arias A.M. (2018). Multi-axial self-organization properties of mouse embryonic stem cells into gastruloids. Nature.

[bib5] Brandenberg N., Hoehnel S., Kuttler F., Homicsko K., Ceroni C., Ringel T., Gjorevski N., Schwank G., Coukos G., Turcatti G., Lutolf M.P. (2020). High-throughput automated organoid culture via stem-cell aggregation in microcavity arrays. Nat. Biomed. Eng..

[bib6] Brower K., Puccinelli R.R., Markin C.J., Shimko T.C., Longwell S.A., Cruz B., Gomez-Sjoberg R., Fordyce P.M. (2018). An open-source, programmable pneumatic setup for operation and automated control of single- and multi-layer microfluidic devices. HardwareX.

[bib7] Buecker C., Srinivasan R., Wu Z., Calo E., Acampora D., Faial T., Simeone A., Tan M., Swigut T., Wysocka J. (2014). Reorganization of enhancer patterns in transition from naive to primed pluripotency. Cell Stem Cell.

[bib8] Cao L., Zhang X., Grimley A., Lomasney A.R., Roper M.G. (2010). Microfluidic multi-analyte gradient generator. Anal. Bioanal. Chem..

[bib9] de Almeida Monteiro Melo Ferraz M., Nagashima J.B., Venzac B., Le Gac S., Songsasen N. (2020). 3D printed mold leachates in PDMS microfluidic devices. Sci. Rep..

[bib10] Deluz C., Friman E.T., Strebinger D., Benke A., Raccaud M., Callegari A., Leleu M., Manley S., Suter D.M. (2016). A role for mitotic bookmarking of SOX2 in pluripotency and differentiation. Genes Dev..

[bib11] Gómez-Sjöberg R., Leyrat A.A., Pirone D.M., Chen C.S., Quake S.R. (2007). Versatile, fully automated, microfluidic cell culture system. Anal. Chem..

[bib12] Guiet R., Burri O., Girgin M.U., Lutolf M. (2021).

[bib13] Gunne-Braden A., Sullivan A., Gharibi B., Sheriff R.S.M., Maity A., Wang Y.-F., Edwards A., Jiang M., Howell M., Goldstone R. (2020). GATA3 mediates a fast, irreversible commitment to BMP4-driven differentiation in human embryonic stem cells. Cell Stem Cell.

[bib14] Hayashi K., Ohta H., Kurimoto K., Aramaki S., Saitou M. (2011). Reconstitution of the mouse germ cell specification pathway in culture by pluripotent stem cells. Cell.

[bib15] Junkin M., Kaestli A.J., Cheng Z., Jordi C., Albayrak C., Hoffmann A., Tay S. (2016). High-content quantification of single-cell immune dynamics. Cell Rep..

[bib16] Kalkan T., Olova N., Roode M., Mulas C., Lee H.J., Nett I., Marks H., Walker R., Stunnenberg H.G., Lilley K.S. (2017). Tracking the embryonic stem cell transition from ground state pluripotency. Development.

[bib17] Kim J., Kang M., Jensen E.C., Mathies R.A. (2012). Lifting gate polydimethylsiloxane microvalves and pumps for microfluidic control. Anal. Chem..

[bib18] Kim J., Koo B.-K., Knoblich J.A. (2020). Human organoids: model systems for human biology and medicine. Nat. Rev. Mol. Cell Biol..

[bib19] Masters J.R., Stacey G.N. (2007). Changing medium and passaging cell lines. Nat. Protoc..

[bib20] Mondragón-Palomino O., Danino T., Selimkhanov J., Tsimring L., Hasty J. (2011). Entrainment of a population of synthetic genetic oscillators. Science.

[bib21] Mulas C., Kalkan T., von Meyenn F., Leitch H.G., Nichols J., Smith A. (2019). Defined conditions for propagation and manipulation of mouse embryonic stem cells. Development.

[bib22] Murakami K., Günesdogan U., Zylicz J.J., Tang W.W.C., Sengupta R., Kobayashi T., Kim S., Butler R., Dietmann S., Azim Surani M. (2016). NANOG alone induces germ cells in primed epiblast in vitro by activation of enhancers. Nature.

[bib23] Niepel M., Hafner M., Mills C.E., Subramanian K., Williams E.H., Chung M., Gaudio B., Barrette A.M., Stern A.D., Hu B. (2019). A multi-center study on the reproducibility of drug-response assays in mammalian cell lines. Cell Syst..

[bib24] Rossi G., Broguiere N., Miyamoto M., Boni A., Guiet R., Girgin M., Kelly R.G., Kwon C., Lutolf M.P. (2021). Capturing cardiogenesis in gastruloids. Cell Stem Cell.

[bib25] Sanchez P.G.L., Mochulska V., Denis C.M., Mönke G., Tomita T., Tsuchida-Straeten N., Petersen Y., Sonnen K.F., François P., Aulehla A. (2021). Arnold tongue entrainment reveals dynamical principles of the embryonic segmentation clock (preprint). Dev. Biol..

[bib49] Schindelin J., Arganda-Carreras I., Frise E., Kaynig V., Longair M., Pietzsch T., Preibisch S., Rueden C., Saalfeld S., Schmid B. (2012). Fiji: an open-source platform for biological-image analysis. Nat. Methods.

[bib26] Schuster B., Junkin M., Kashaf S.S., Romero-Calvo I., Kirby K., Matthews J., Weber C.R., Rzhetsky A., White K.P., Tay S. (2020). Automated microfluidic platform for dynamic and combinatorial drug screening of tumor organoids. Nat. Commun..

[bib27] Simunovic M., Brivanlou A.H. (2017). Embryoids, organoids and gastruloids: new approaches to understanding embryogenesis. Development.

[bib28] Sonnen K.F., Lauschke V.M., Uraji J., Falk H.J., Petersen Y., Funk M.C., Beaupeux M., François P., Merten C.A., Aulehla A. (2018). Modulation of phase shift between Wnt and notch signaling oscillations controls mesoderm segmentation. Cell.

[bib29] Sorre B., Warmflash A., Brivanlou A.H., Siggia E.D. (2014). Encoding of temporal signals by the TGF-β pathway and implications for embryonic patterning. Dev. Cell.

[bib30] Steventon B., Busby L., Arias A.M. (2021). Establishment of the vertebrate body plan: rethinking gastrulation through stem cell models of early embryogenesis. Dev. Cell.

[bib31] Strawbridge S.E., Blanchard G.B., Smith A., Kugler H., Martello G. (2020). Embryonic stem cells commit to differentiation by symmetric divisions following a variable lag period (preprint). Dev. Biol..

[bib32] Thorsen T., Maerkl S.J., Quake S.R. (2002). Microfluidic large-scale integration. Science.

[bib33] Tischler J., Gruhn W.H., Reid J., Allgeyer E., Buettner F., Marr C., Theis F., Simons B.D., Wernisch L., Surani M.A. (2019). Metabolic regulation of pluripotency and germ cell fate through α-ketoglutarate. EMBO J..

[bib34] Turner D.A., Girgin M., Alonso-Crisostomo L., Trivedi V., Baillie-Johnson P., Glodowski C.R., Hayward P.C., Collignon J., Gustavsen C., Serup P. (2017). Anteroposterior polarity and elongation in the absence of extraembryonic tissues and spatially localised signalling in *Gastruloids*, mammalian embryonic organoids. Development.

[bib35] van den Brink S.C., Baillie-Johnson P., Balayo T., Hadjantonakis A.-K., Nowotschin S., Turner D.A., Martinez Arias A. (2014). Symmetry breaking, germ layer specification and axial organisation in aggregates of mouse embryonic stem cells. Development.

[bib36] van den Brink S.C., van Oudenaarden A. (2021). 3D gastruloids: a novel Frontier in stem cell-based in vitro modeling of mammalian gastrulation. Trends Cell Biol..

[bib37] van Oostrom M.J., Meijer W.H.M., Sonnen K.F. (2021). A microfluidics approach for the functional investigation of signaling oscillations governing somitogenesis. JoVE.

[bib38] Vianello S., Girgin M., Rossi G., Lutolf M. (2020).

[bib39] Vianello S., Girgin M., Rossi G., Lutolf M. (2020).

[bib40] Vianello S., Sanchez P.G., Bercowsky-Rama A. (2020).

[bib41] White J.A., Streets A.M. (2018). Controller for microfluidic large-scale integration. HardwareX.

[bib42] Wong B.G., Mancuso C.P., Kiriakov S., Bashor C.J., Khalil A.S. (2018). Precise, automated control of conditions for high-throughput growth of yeast and bacteria with eVOLVER. Nat. Biotechnol..

[bib43] Woodruff K., Maerkl S.J. (2018). Microfluidic module for real-time generation of complex multimolecule temporal concentration profiles. Anal. Chem..

[bib44] Wray J., Kalkan T., Gomez-Lopez S., Eckardt D., Cook A., Kemler R., Smith A. (2011). Inhibition of glycogen synthase kinase-3 alleviates Tcf3 repression of the pluripotency network and increases embryonic stem cell resistance to differentiation. Nat. Cell Biol..

[bib45] Ying Q.-L., Wray J., Nichols J., Batlle-Morera L., Doble B., Woodgett J., Cohen P., Smith A. (2008). The ground state of embryonic stem cell self-renewal. Nature.

[bib46] Zhang C., Tu H.-L., Jia G., Mukhtar T., Taylor V., Rzhetsky A., Tay S. (2019). Ultra-multiplexed analysis of single-cell dynamics reveals logic rules in differentiation. Sci. Adv..

[bib47] Zhang X., Grimley A., Bertram R., Roper M.G. (2010). Microfluidic system for generation of sinusoidal glucose waveforms for entrainment of islets of langerhans. Anal. Chem..

[bib48] Zylicz J.J., Dietmann S., Günesdogan U., Hackett J.A., Cougot D., Lee C., Surani M.A. (2015). Chromatin dynamics and the role of G9a in gene regulation and enhancer silencing during early mouse development. Elife.

