# An automated do-it-yourself system for dynamic stem cell and organoid culture in standard multi-well plates

**DOI:** 10.1016/j.crmeth.2022.100310

**Published:** 2022-10-24

**Authors:** Julia Tischler, Zoe Swank, Hao-An Hsiung, Stefano Vianello, Matthias P. Lutolf, Sebastian J. Maerkl

## Main text

(Cell Reports Methods *2*, 100244-1–100244-14; e1–e6; July 18, 2022)

During figure preparation for this article, the authors accidentally transposed the two image panels of Figure 3E. The authors regret this error and apologize for any confusion that it might have caused. The article has now been corrected online.

**Figure 3E:**
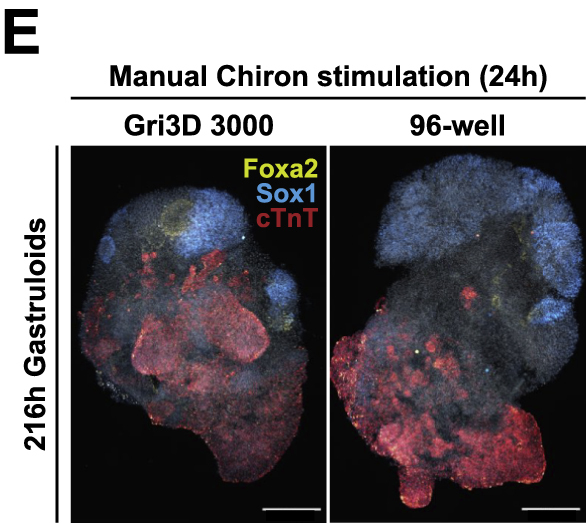
Developmental potential of 3D gastruloids in response to time-varying Chir stimulation (corrected)

**Figure 3E:**
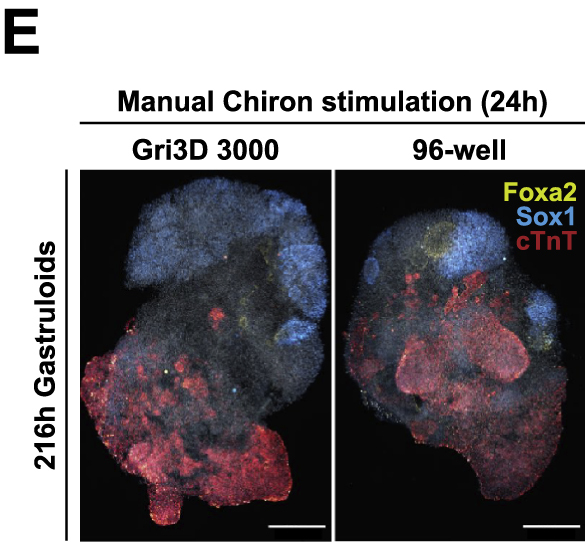
Developmental potential of 3D gastruloids in response to time-varying Chir stimulation (original)

