# Supplementary material for: An automated do-it-yourself system for dynamic stem cell and organoid culture in standard multi-well plates
Source: Cell Rep Methods. 2022 Jul 1;2(7):100244. doi: 10.1016/j.crmeth.2022.100244 (PMC9308133; doi:10.1016/j.crmeth.2022.100244)
Supplement: Document S1. Figures S1–S3 [file mmc1.pdf]

Cell Reports Methods, Volume *2*

Supplemental information

An automated do-it-yourself system for dynamic stem cell and organoid culture in standard multi-well plates

Julia Tischler, Zoe Swank, Hao-An Hsiung, Stefano Vianello, Matthias P. Lutolf, and Sebastian J. Maerkl

Supplemental Information

Figures S1 – S3

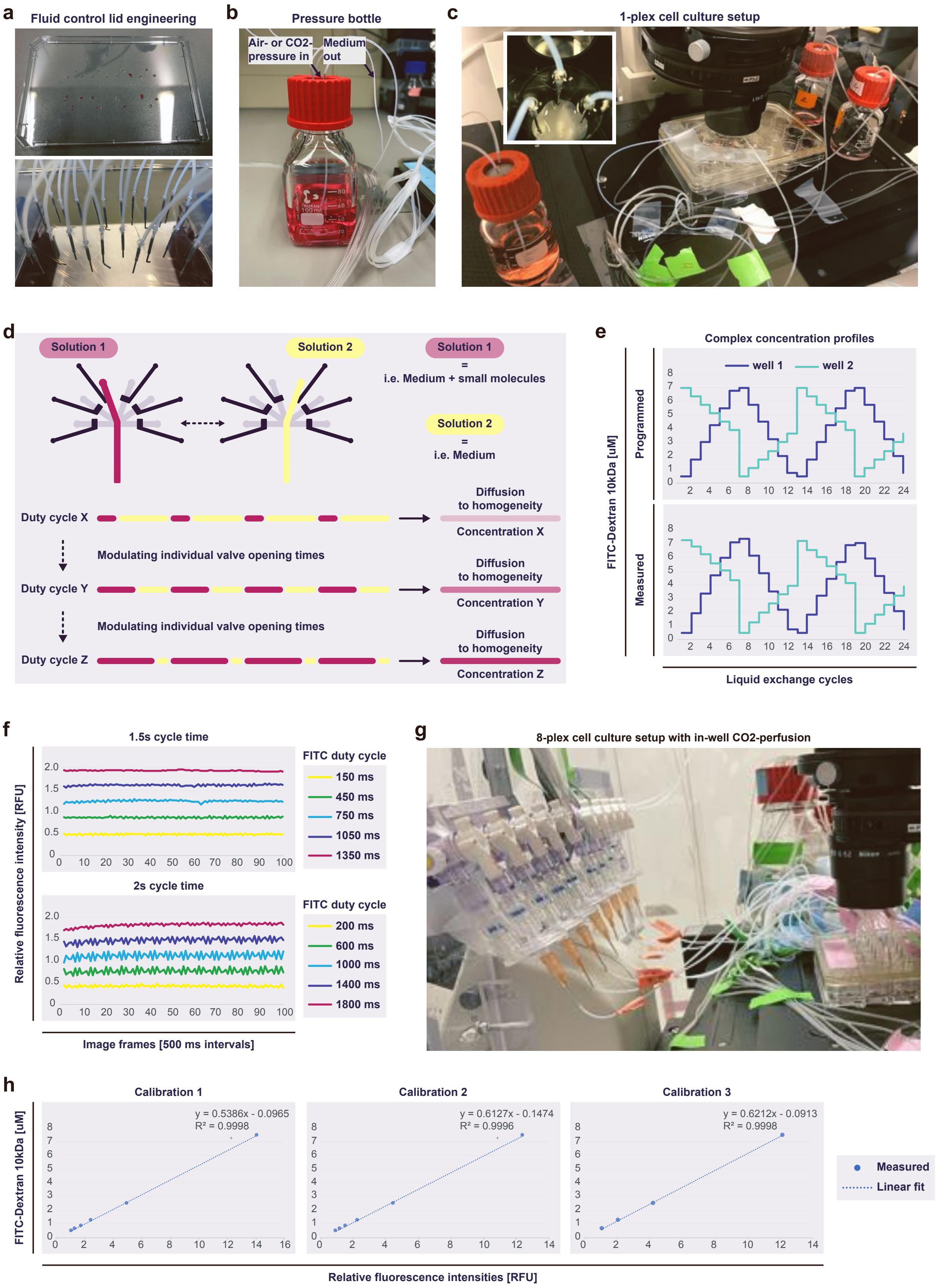

a
Fluid control lid engineering
b
Pressure bottle
Air- or CO2-
pressure in
Medium
out
c
1-plex cell culture setup
d
Solution 1
Solution 2
Solution 1
=
i.e. Medium + small molecules
Solution 2
=
i.e. Medium
Duty cycle X
Diffusion
to homogeneity
Concentration X
Modulating individual valve opening times
Duty cycle Y
Diffusion
to homogeneity
Concentration Y
Modulating individual valve opening times
Duty cycle Z
Diffusion
to homogeneity
Concentration Z
e
Complex concentration profiles
well 1
well 2
FITC-Dextran 10kDa [uM]
Programmed
Measured
Liquid exchange cycles
f
1.5s cycle time
FITC duty cycle
150 ms
450 ms
750 ms
1050 ms
1350 ms
2s cycle time
FITC duty cycle
200 ms
600 ms
1000 ms
1400 ms
1800 ms
Relative fluorescence intensity [RFU]
Image frames [500 ms intervals]
g
8-plex cell culture setup with in-well CO2-perfusion
h
Calibration 1
y = 0.5386x - 0.0965
R² = 0.9998
Calibration 2
y = 0.6127x - 0.1474
R² = 0.9996
Calibration 3
y = 0.6212x - 0.0913
R² = 0.9998
FITC-Dextran 10kDa [uM]
Measured
Linear fit
Relative fluorescence intensities [RFU]

Figure S1. DIY platform for the fully automated culture of mammalian cells, related to Figure 1

(a) Building the fluidic control lid. Standard polystyrene lids are pierced with three holes per individual well, and fitted with tubing for medium inflow, outflow, and level setting, entering culture chambers from the top. Metal pins inserted at the medium-facing end of the fluidic interconnects provide additional stability, and enable rapid re-adjustments of the culture volumes. (b) Pressure-driven medium inflow bottle. (c) Setup for single-well cell culture on the ACCP. (d) Schematic representing the PWM-mediated formulation of time-varying concentration profiles. (e) Complex concentration profiles, generated on the ACCP through PWM-mediated mixing of a 7.5µM fluoresceinisothiocyanat (FITC)-dextran 10kDa solution and medium, and dispensing into two parallel culture wells. (f) Determining the homogeneity of output solutions exiting a flow path of 37.5 cm (PTFE tubing, with an inner diameter of 0.56 mm), following the PWM-mediated mixing of a 7.5µM FITC-dextran 10kDa solution and buffer, at an inflow pressure of 10 psi. Images were acquired with a time-resolution of 500ms. Relative median fluorescence intensities are shown. (g) Setup for the culture of 3D gastruloids in hydrogel microwell arrays on the ACCP, with direct in-well CO_2 perfusion. (h) Calibration curves derived from linear fits through relative median fluorescence intensity measurements as a function of known FITC-dextran 10kDa concentrations.

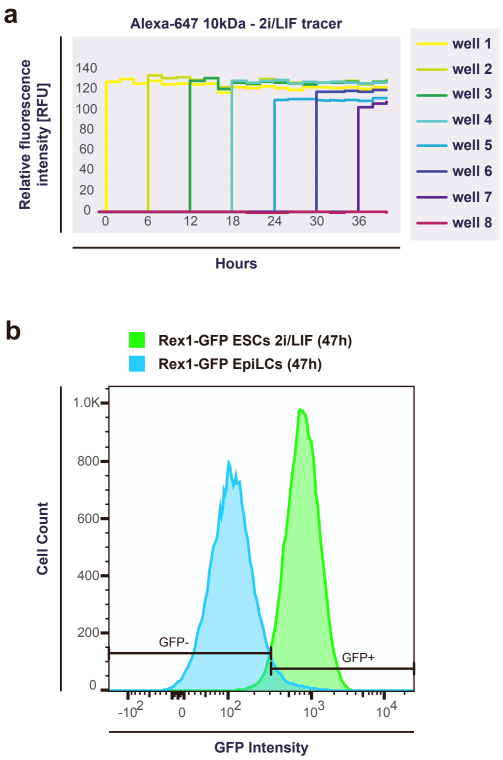

Figure S2. Tracking cellular commitment along the developmental trajectory toward epiblast fate, related to Figure 2

(a) Alexa Fluor 647-dextran 10kDa tracing 2i/LIF medium in automated medium-switch experiments during the ESC-to-EpiLC conversion, performed on the ACCP. Relative median fluorescence intensities are shown. (b) Overlay of flow cytometer profiles of ESCs cultured in 2i/LIF conditions and EpiLCs following 47h of stimulation used to determine the cut-off for *Rex1*-GFP positive (GFP+) and negative (GFP-) populations.

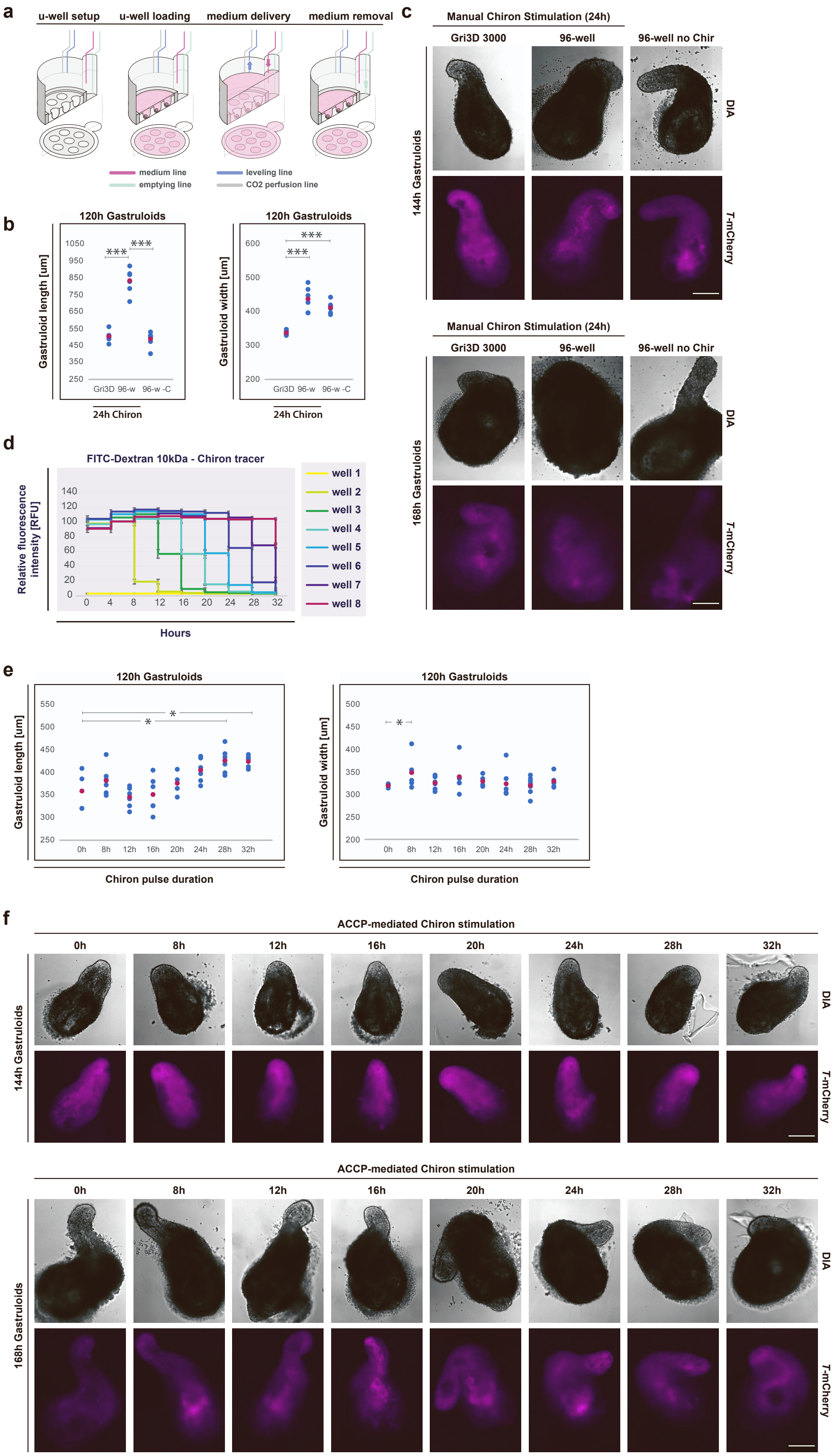
a
u-well setup
u-well loading
medium delivery
medium removal
medium line
leveling line
emptying line
CO2 perfusion line
b
120h Gastruloids
Gastruloid length [um]
1050
950
850
750
650
550
450
350
250
***
***
Gri3D 96-w 96-w -C
24h Chiron
120h Gastruloids
Gastruloid width [um]
600
500
400
300
200
***
***
Gri3D 96-w 96-w -C
24h Chiron
c
Manual Chiron Stimulation (24h)
Gri3D 3000
96-well
96-well no Chir
144h Gastruloids
DIA
T-mCherry
Manual Chiron Stimulation (24h)
Gri3D 3000
96-well
96-well no Chir
168h Gastruloids
DIA
T-mCherry
d
FITC-Dextran 10kDa - Chiron tracer
Relative fluorescence intensity [RFU]
140
120
100
80
60
40
20
0
0 4 8 12 16 20 24 28 32
Hours
well 1
well 2
well 3
well 4
well 5
well 6
well 7
well 8
e
120h Gastruloids
Gastruloid length [um]
550
500
450
400
350
300
250
*
*
0h 8h 12h 16h 20h 24h 28h 32h
Chiron pulse duration
120h Gastruloids
Gastruloid width [um]
500
450
400
350
300
250
200
*
0h 8h 12h 16h 20h 24h 28h 32h
Chiron pulse duration
f
ACCP-mediated Chiron stimulation
0h 8h 12h 16h 20h 24h 28h 32h
144h Gastruloids
DIA
T-mCherry
ACCP-mediated Chiron stimulation
0h 8h 12h 16h 20h 24h 28h 32h
168h Gastruloids
DIA
T-mCherry

Figure S3. Developmental potential of 3D gastruloids cultured in Gri3D hydrogel microwell arrays, related to Figure 3

(a) Schematic of Gri3D 3000 hydrogel microwell arrays (SUN bioscience). A medium reservoir adjacent to the aggregate-containing microwell arrays enables near-complete medium exchanges without impacting cellular aggregates. (b, c) Gastruloid development in Gri3D microwell arrays and low-adhesion 96-well plates, respectively. Gastruloids were assembled from *Sox1*-GFP::*Brachyury*-mCherry (*SBr*) reporter ESCs. (b) 120h gastruloids, with mean gastruloid length and width depicted in magenta. Gri3D, n=7; 96-w, 96-well, n=6; 96-w -C, 96-well, without Chir stimulation, n=7. ***, $p \leq 0.005$ (unpaired 1-tailed Student's *t*-test). (d) FITC-dextran 10kDa tracking N2B27 medium supplemented with Chir in time-varying Chir pulse experiments performed on the ACCP. Averages of relative median fluorescence intensities across four individual microwells are shown. Error bars represent ± SD. (e, f) Developmental potential of *SBr* reporter gastruloids in Gri3D microwell arrays, following time-varying Chir stimulation on the ACCP. (e) 120h gastruloids, mean gastruloid length and width are indicated in magenta. 0h, n=4; 8h, n=6; 12h, n=6; 16h, n=6; 20h, n=4; 24h, n=6; 28h, n=7; 32h, n=5. *, $p \leq 0.05$ (unpaired 1-tailed Student's *t*-test). (c, f) Gastruloids shown are later developmental stages of the matching gastruloids depicted in Figure 3b and g, respectively. Scalebars, 250µm. Chir, Chiron. -C, without Chiron stimulation.
